# Eutectic‐Like Ion‐Conductive Phase‐Incorporated Zwitterionic Covalent Organic Framework Solid Electrolyte for All‐Solid‐State Li Metal Batteries

**DOI:** 10.1002/advs.202505530

**Published:** 2025-06-25

**Authors:** Jaewoo Lee, Jae‐Hoon Shin, Sungpyo Hong, Jun‐Hyeong Lee, Dae‐Hui Jeong, Hongwon Kim, Yong Hui Kim, Sang Uck Lee, Jong‐Ho Kim

**Affiliations:** ^1^ Department of Materials Science and Chemical Engineering Hanyang University Ansan 15588 Republic of Korea; ^2^ School of Chemical Engineering Sungkyunkwan University Suwon 16149 Republic of Korea

**Keywords:** All‐solid‐state Li metal battery, covalent organic framework, dendrite suppression, eutectic ion channel, zwitterionic solid electrolyte

## Abstract

High energy‐density Li metal batteries with improved safety and cyclic performance can be achieved by adopting solid polymer electrolytes. However, current solid polymer electrolytes show inferior room‐temperature ionic conductivity and Li‐ion transference number (t_Li+_), impeding their practical applications. Herein, a 3‐(pyridinium)propane‐1‐sulfonate zwitterionic covalent organic framework (PSZ‐COF) incorporating a eutectic‐like ion‐conductive phase is prepared by making a complex with *N*‐methyl‐*N*‐propylpyrrolidinium bis(fluorosulfonyl)imide (Pyrrol‐FSI) and Li salts in its channels as a solid electrolyte (eutectic PSZ‐COF). The eutectic PSZ‐COF possesses a zwitterionic Li‐salt‐dissociating mode, a Pyrrol‐FSI Li‐cation‐transporting carrier, and a pyridinium anion‐holding trap in the ordered channels, providing a high ionic conductivity (0.127 mS cm^−1^) and t_Li+_ (0.62) at room temperature. Moreover, the eutectic PSZ‐COF can suppress Li dendrites and drive a LiF/Li_3_N‐rich solid electrolyte interface layer on a Li anode. Computational simulations reveal that the time‐evolved average distance and hoping distance of Li^+^ ions are significantly reduced in the eutectic PSZ‐COF, allowing their facile migration. The all‐solid‐state Li metal batteries bearing eutectic PSZ‐COF with a lithium iron phosphate(LFP) cathode (2.0 mg cm^−^^2^) exhibit an initial capacity of 153.3 mAh g^−1^, 99.9% Coulombic efficiency, and 100% capacity retention over 150 cycles at room temperature. This study offers an effective route to high‐performance solid electrolytes for all‐solid‐state Li batteries.

## Graphical abstract

The eutectic‐like pyridinium sulfonate zwitterionic (PSZ) COF solid electrolyte incorporating a Li salt‐dissociating mode, a Li^+^ cation‐transporting carrier, and an anion‐holding trap is designed for all‐solid‐state Li metal batteries. The eutectic PSZ‐COF solid electrolyte exhibits superior Li‐ion conductivity and transference number at room temperature along with effective suppression of Li dendrites, leading to the high performance of the all‐solid‐state batteries.

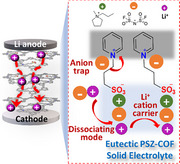

## 1 Introduction

The demand for high energy‐density Li batteries keeps growing due to the rapid expansion of the electric vehicle market as well as climate change concerns.^[^ ^1^ ^]^ High energy‐density Li batteries can be designed by replacing a graphite anode with a Li metal that has a high theoretical capacity (3860 mAh g^−1^) and a low electrochemical potential (−3.04 V vs SHE).^[^ ^2^ ^]^ The use of this Li metal anode for high energy‐density Li metal batteries essentially requires the development of proper electrolytes that allow the stable stripping and plating of Li ions on the reactive anode for their long‐term cycle.^[^ ^3^ ^]^ Conventional organic liquid electrolytes, however, cannot suppress the uncontrolled formation of lithium dendrites and solid electrolyte interface (SEI) layers on the anode of Li metal batteries, leading to acceleration of cell fading.^[^ ^4^ ^]^ Furthermore, conventional organic liquid electrolytes exhibit poor thermal and electrochemical stabilities, and high explosion peril in Li metal batteries.^[^ ^5^ ^]^ Thereby, the discovery of solid‐state electrolytes that are compatible with a Li metal anode as well as high‐capacity cathode materials is of great significance to provide high energy‐density Li metal batteries.^[^ ^6^ ^]^

Solid polymer electrolytes have been considered promising solid electrolytes suitable for realizing high energy‐density Li metal batteries through suppression of uncontrollable dendrites, formation of conductive SEI layers, and reduction of the fire risk.^[^ ^7^ ^]^ Moreover, solid polymer electrolytes display better electrode contact with minimizing voids and cracks than inorganic solid electrolytes, being beneficial for the effective suppression of dendrites and dead Li.^[^ ^8^ ^]^ Solid polymer electrolytes also offer easier processibility in the industrial fabrication of all‐solid‐state Li metal batteries. To date, extensive efforts have been made on the development of poly(ethylene oxide) (PEO) and PEO copolymer‐based solid electrolytes for Li metal batteries.^[^ ^9^ ^]^ PEO‐based solid electrolytes, however, have shown poor ionic conductivity and a low Li‐ion transference number at room temperature, impeding their commercial application for all‐solid‐state Li metal batteries.^[^ ^10^ ^]^ The insufficient room‐temperature ionic conductivity and Li‐ion transference number of PEO‐based solid electrolytes can be attributed to the inactive segmental motion of polymer chains,^[^ ^11^ ^]^ the poor dissociation of Li salts, and the random formation of ionic transport channels at room temperature.^[^ ^12^ ^]^ To overcome the limitations of these PEO‐based solid electrolytes, non‐PEO‐based solid polymer electrolytes, including solid molecular ionic composites,^[^ ^7b^ ^]^ polymeric ionic liquids^[^ ^6^, ^13^ ^]^ and elastomeric polymers,^[^ ^14^ ^]^ have been reported for their applications to all‐solid‐state Li metal batteries. Despite these remarkable contributions, new strategies for designing solid polymer electrolytes that can offer high ionic conductivity, superior Li‐ion transference number, and ordered ionic channels at room temperature are required for all‐solid‐state Li metal batteries with high energy density.

Covalent organic frameworks (COFs) are crystalline polymers with well‐ordered pore structures, which can be prepared readily by strong covalent bonds of organic building blocks.^[^ ^15^ ^]^ The pore size and chemical functionality of COFs can be precisely controlled by varying diverse building units in simple organic reactions.^[^ ^16^ ^]^ The facile structural and functional tunability of COFs have driven development of COF‐based solid electrolytes,^[^ ^17^ ^]^ including PEO‐introduced COFs,^[^ ^18^ ^]^ anionic COFs,^[^ ^19^ ^]^ cationic COFs,^[^ ^20^ ^]^ COF‐embedded ionic liquids,^[^ ^21^ ^]^ and zwitterionic COFs.^[^ ^22^ ^]^ Each of the COF solid electrolytes offers a unique strategy for Li‐ion conduction, showing improved performance in Li metal batteries. Some of the previous COF electrolytes contained a large portion of additives that were predominant ion conductors, resulting in quasi solid‐state electrolytes. An ideal form of COF solid electrolytes needs to possess a Li salt‐dissociating mode, a Li cation‐transporting carrier, an anion‐holding trap, and an ordered ionic channel to achieve both superior ionic conductivity and Li‐ion transference number at room temperature. Devising an effective strategy for structuring the above requirements in COF solid electrolytes remains a challenge and still requires extensive investigations. In particular, the fundamental mechanism capable of facile Li‐salt dissociation and fast Li‐cation dynamics in COF solid electrolytes needs to be more deeply investigated for all‐solid‐state Li metal batteries, since a few studies were reported.^[^ ^19e^ ^]^

Herein, the 3‐(pyridinium)propane‐1‐sulfonate zwitterionic (PSZ) COF bearing a eutectic‐like ion‐conductive phase (denoted “eutectic PSZ‐COF”) was prepared as a solid electrolyte for all‐solid‐state Li metal batteries by incorporating a small portion of *N*‐methyl‐*N*‐propylpyrrolidinium bis(fluorosulfonyl)imide (Pyrrol‐FSI) into the channels of pristine PSZ‐COF along with lithium bis(fluorosulfonyl)imide (LiFSI) as a Li salt. The eutectic‐like complex among the zwitterions, Pyrrol‐FSI, and LiFSI in the ordered channels of the eutectic PSZ‐COF solid electrolyte served as a Li salt‐dissociating moiety and a Li cation‐transporting carrier, while the cationic pyridinium groups of the PSZ‐COF plane performed as anion‐holding traps. The chemical and physical structures of the eutectic PSZ‐COF solid electrolyte were thoroughly investigated by ^7^Li nuclear magnetic resonance (NMR) spectroscopy, Fourier‐transform infrared (FT‐IR) spectroscopy, X‐ray photoelectron spectroscopy (XPS), and transmission electron microscopy (TEM). In addition, the electrochemical properties of the eutectic PSZ‐COF solid electrolyte were fully examined, including Li‐ion conduction, dendrite suppression, and SEI formation. The effective Li‐ion dissociation and diffusion dynamics in the eutectic PSZ‐COF solid electrolyte were also understood by density functional theory (DFT) and molecular dynamics (MD) simulations. The eutectic PSZ‐COF solid electrolyte was then applied to all‐solid‐state Li metal batteries with a cathode of LiFePO_4_ (LFP) or LiNi_6_Co_2_Mn_2_O_2_ (NCM_622_), showing high specific capacities and Coulombic efficiencies.

## 2 Results and Discussion

### 2.1 Synthesis and Characterization of PSZ‐COF

**Figure 1:**
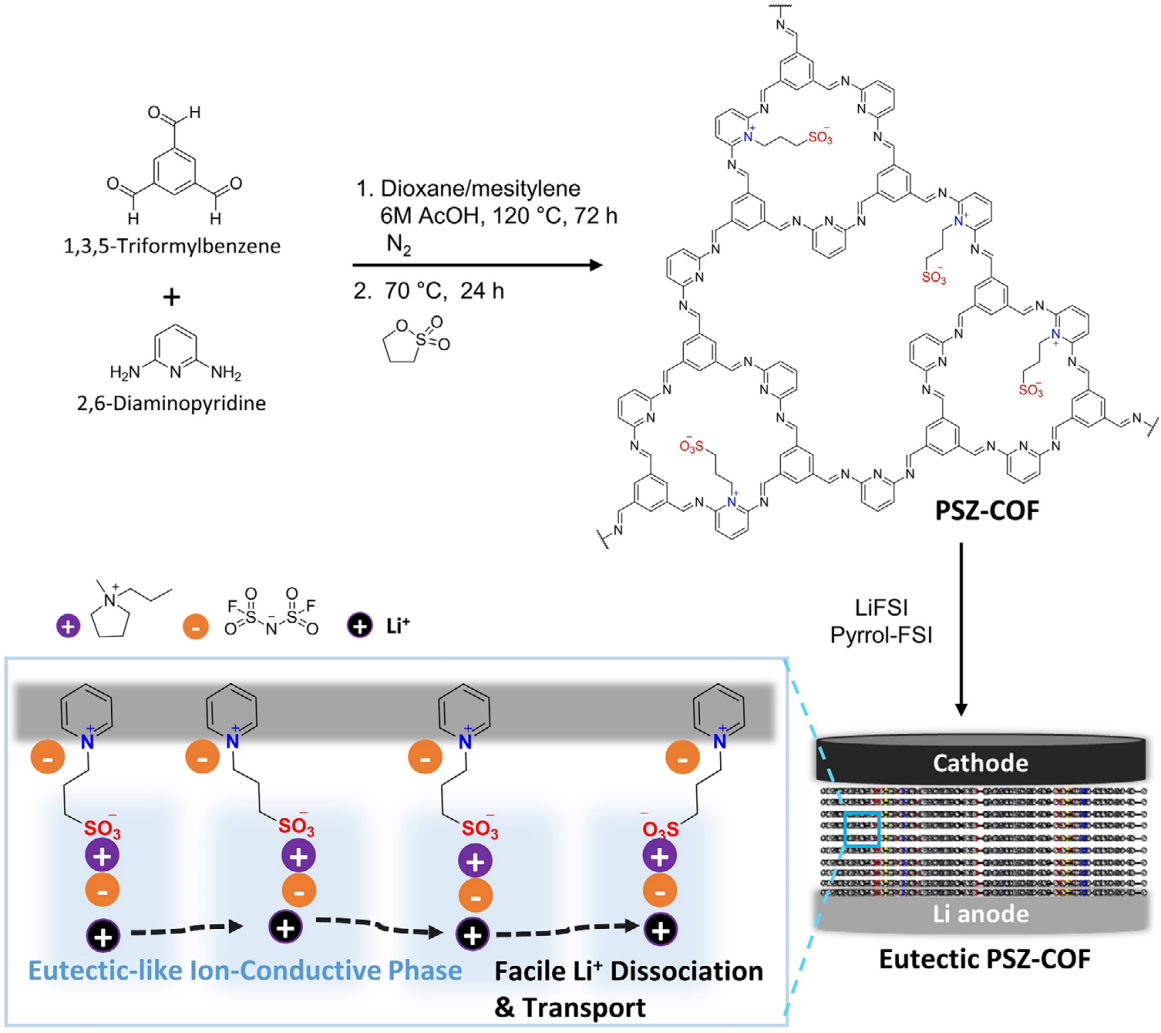
Schematic illustration for the synthesis of 3‐(pyridinium)propane‐1‐sulfonate zwitterionic covalent organic framework (PSZ‐COF) solid electrolyte with a eutectic‐like ion‐conductive complex with *N*‐methyl‐*N*‐propylpyrrolidinium bis(fluorosulfonyl)imide (Pyrrol‐FSI) and LiFSI in a solid electrolyte (Eutectic PSZ‐COF) for all‐solid‐state Li metal batteries.

Eutectic PSZ‐COF solid electrolyte was prepared in simple solution reactions (**Figure** **1**). Pyridine‐rich COF (PCOF) was first obtained by the Schiff base reaction of 1,3,5‐triformylbenzene and 2,6‐diaminopyridine under acidic conditions. Then, PCOF reacted with 1,3‐propanesultone to introduce the zwitterionic groups (PSZ) on its pyridine sites, resulting in the formation of pristine PSZ‐COF. Finally, Pyrrol‐FSI was incorporated into the pristine PSZ‐COF along with Li salts (LiFSI) to produce the eutectic PSZ‐COF solid electrolyte. In the eutectic PSZ‐COF solid electrolyte, the interaction of the zwitterionic groups with both Pyrrol‐FSI and LiFSI yielded the eutectic‐like ion‐conductive phase in the pores as depicted in Figure 1. Specifically, the eutectic PSZ‐COF solid electrolyte consisted of a Li salt‐dissociating mode of zwitterionic groups, a Li cation‐transporting carrier of Pyrrol‐FSI, and an anion‐holding trap of cationic pyridinium groups. The eutectic‐like ion‐conductive phase of the PSZ‐COF solid electrolyte in the ordered channels could facilitate Li‐ion dissociation and transport at room temperature.

**Figure 2:**
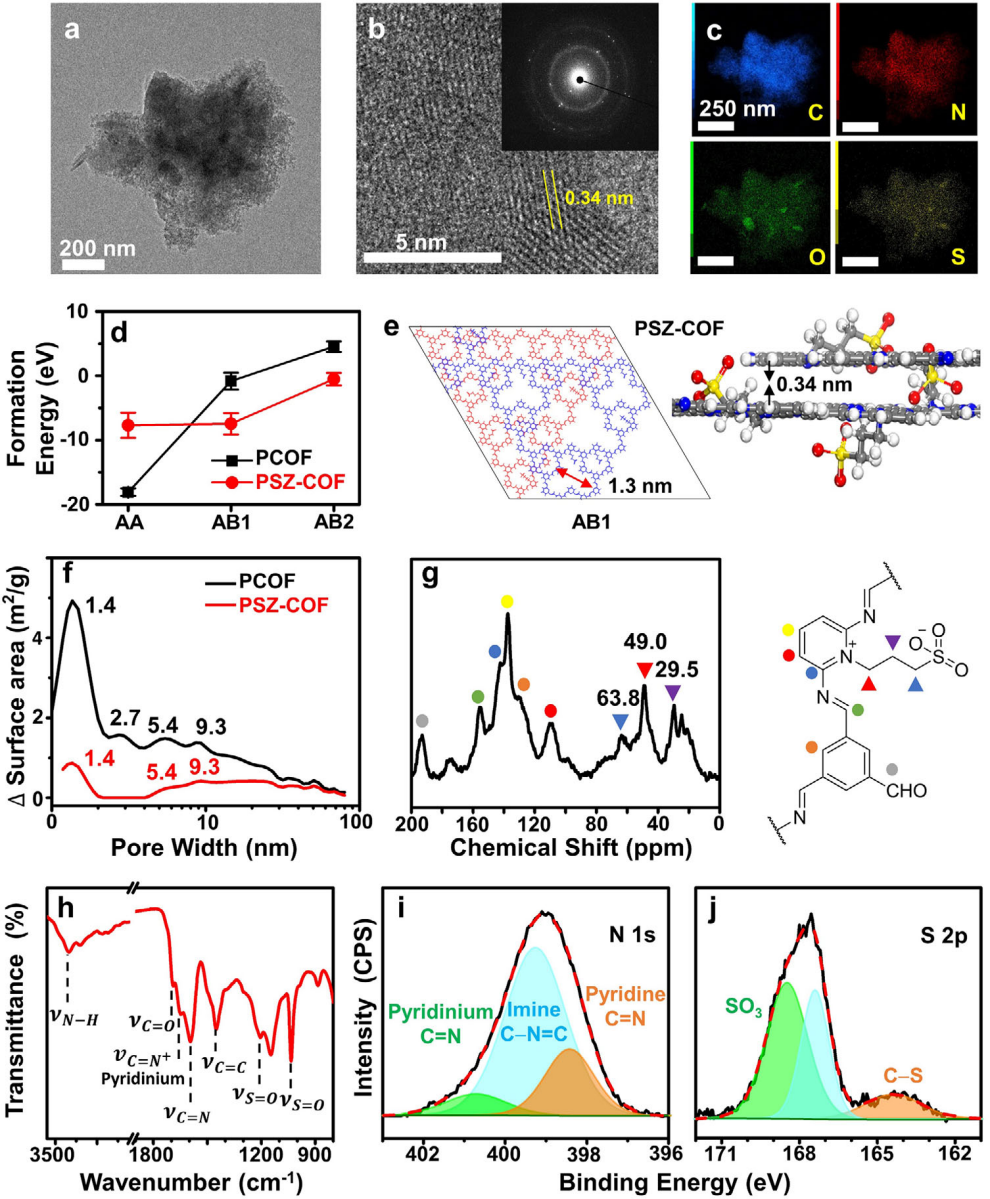
Physical and chemical structures of pristine PSZ‐COF. a,b) HR‐TEM images of PSZ‐COF. Inset shows its SAED pattern. c) EDS atomic mapping images of PSZ‐COF. d) Formation energies for the possible stacking structures of PSZ‐COF and PCOF. e) AB1 stacking structure of PSZ‐COF with a corresponding d‐spacing, where the blue and red layers represent its repeating layer structures in the AB1. f) Pore size distribution of PCOF and PSZ‐COF. g) Solid‐state ^13^C NMR spectrum of PSZ‐COF. h) FT‐IR spectrum of PSZ‐COF. i) N 1s and j) S2p XPS spectra of PSZ‐COF.

The physical and chemical structures of pristine PSZ‐COF were first characterized as shown in **Figure** **2**. The HR‐TEM images of pristine PSZ‐COF revealed that it had a crystalline stacking morphology with an interlayer spacing of 0.34 nm (Figure 2a,b). The energy dispersive X‐ray spectrometry (EDS) elemental mapping of pristine PSZ‐COF clearly confirmed the uniform distribution of C, N, O, and S atoms on its skeleton (Figure 2c), indicating that the zwitterionic groups were evenly incorporated throughout the COF plane. The content of the zwitterionic groups in the pristine PSZ‐COF was quantified by elemental analysis based on combustion (Table S1, Supporting Information), showing that 30% of the initial pyridine sites were converted into the 3‐(pyridinium)propane‐1‐sulfonate zwitterions.

The stacking structures of the pristine PSZ‐COF were then investigated through simulations and experimental analyses. As illustrated in Figure S1 (Supporting Information), three possible stacking structures (AA, AB1, and AB2) of the pristine PSZ‐COF as well as PCOF can be designed based on stacking sequences. In the AA configuration, the layers are eclipsed directly on top of each other in the same plane. In contrast, the AB1 and AB2 configurations have layers staggered from each other with different degrees of lateral displacement. The formation energies for all these stacking structures were then calculated. The results implied that the AA and AB1 sequences were equally favorable for the pristine PSZ‐COF, while the AA configuration was most stable for PCOF (Figure 2d). The interlayer spacing optimization of the stacking structures confirmed that the interlayer spacing of the AB stacking configurations of the pristine PSZ‐COF was 0.3 nm smaller than that of its AA stacking, while PCOF showed a smaller difference in the interlayer spacing of AA and AB (Figure 2e; Figures S3 and S4, Supporting Information). The mitigated steric hindrance between the zwitterionic groups in the AB1 configuration of PSZ‐COF gave rise to a stability nearly comparable to that of the AA stacking. The XRD pattern of the pristine PSZ‐COF displayed the broadening of the peak for the (0 0 1) plane with an interlayer spacing of 0.34 nm, whereas PCOF showed characteristic peaks at 3.10°, 7.35°, and 23.1° for the (1 0 0), (2 0 0), and (0 0 1) planes, respectively (Figures S5 and S6, Supporting Information). Although the crystallinity of the pristine PSZ‐COF appeared to be reduced in the XRD spectrum after incorporation of the zwitterionic groups, its HR‐TEM image in Figure 2b revealed that it retained a periodic crystalline structure. The Brunauer–Emmett–Teller (BET) pore distribution also found that the pristine PSZ‐COF retained a pore size of 1.4 nm (Figure 2f; Figure S7, Supporting Information), which was in good agreement with the characteristic pore size (1.3 nm) of the AB1 configuration with an interlayer spacing of 0.34 nm. All these experimental and simulation results suggested that the incorporation of the zwitterionic groups did not change the intrinsic hexagonal pore structure of the pristine PSZ‐COF.

The chemical structure of the pristine PSZ‐COF was further confirmed by solid‐state ^13^C‐NMR, FT‐IR, and XPS. The ^13^C‐NMR spectrum of PSZ‐COF displayed the characteristic peaks for the 3‐(pyridinium)propane‐1‐sulfonate zwitterions at 63.8, 49.0, and 29.5 ppm (Figure 2g), which were not observed in the spectrum of PCOF (Figure S8, Supporting Information). The stretching vibrational modes of pyridinium C═N^+^ and sulfonate S═O bonds for the zwitterionic groups clearly appeared at 1648 and 1206, and 1034 cm^−1^, respectively, in the FT‐IR spectrum of the pristine PSZ‐COF (Figure 2h), while no peaks were observed in that of PCOF (Figure S9, Supporting Information). The presence of the zwitterionic groups in PSZ‐COF was further identified in its XPS spectrum. The N 1s XPS spectrum of the pristine PSZ‐COF displayed a clear peak of pyridinium C═N at 400.7 eV along with imine and pyridine peaks at 399.3 and 398.4 eV (Figure 2i).^[^ ^23^ ^]^ Moreover, the sulfonate and C−S bonds of the zwitterionic groups newly appeared at 168.5 and 164.4 eV in the S 2p XPS spectrum of PSZ‐COF (Figure 2j),^[^ ^24^ ^]^ whereas no peaks were observed in the spectra of PCOF (Figures S10–S12, Supporting Information). These spectroscopic analytical results revealed that the zwitterionic groups were successfully introduced to the pyridinic N sites to form the desired PSZ‐COF.

### 2.2 Confirmation of Eutectic‐Like Ion‐Conductive Phase in Eutectic PSZ‐COF Solid Electrolyte

**Figure 3:**
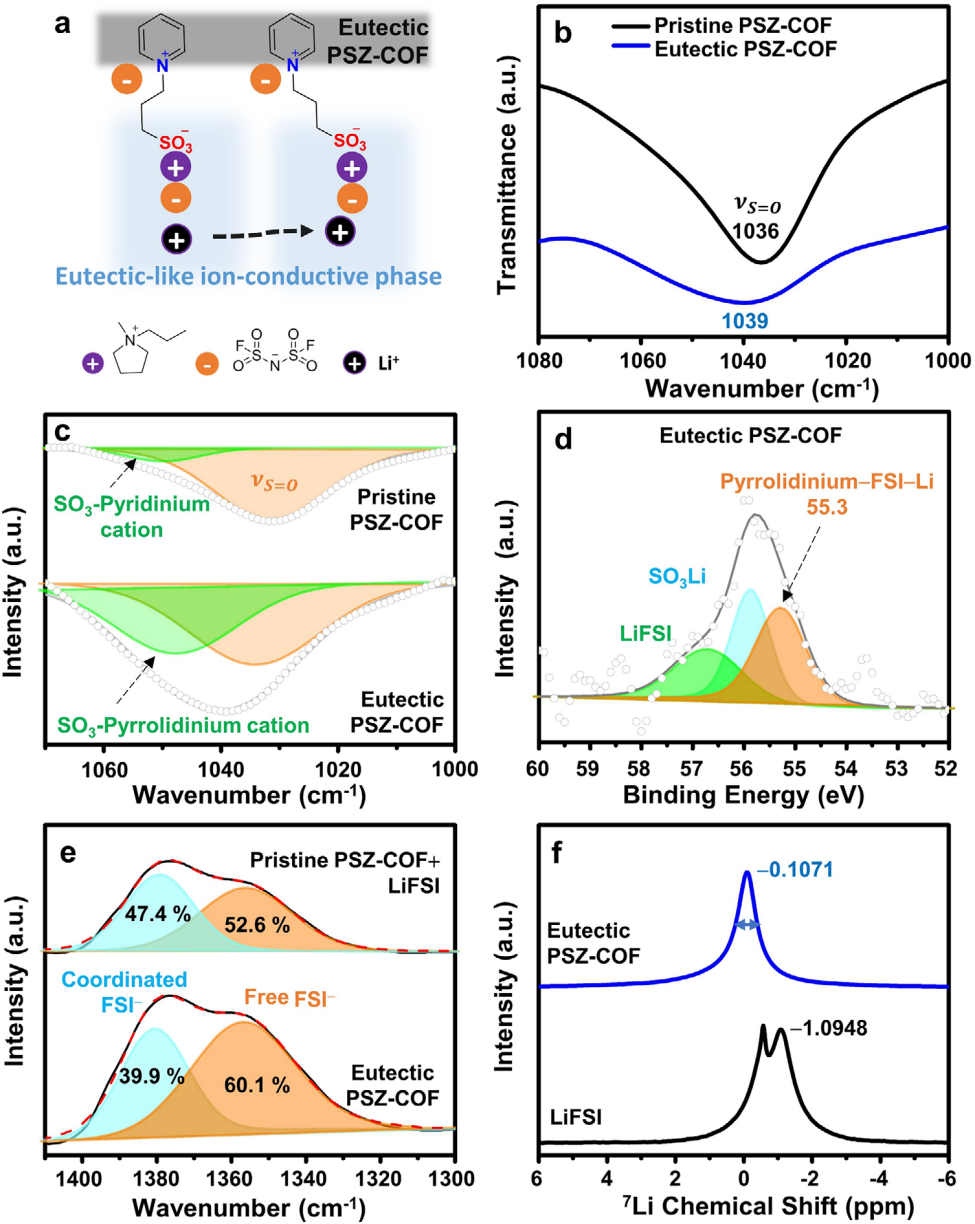
Confirmation of eutectic‐like ion‐conductive phase in eutectic PSZ‐COF solid electrolyte. a) Schematic illustration of the formation of the eutectic‐like ion‐conductive phase in the eutectic PSZ‐COF solid electrolyte. b) FT‐IR spectra of pristine PSZ‐COF and the eutectic PSZ‐COF solid electrolyte with a eutectic‐like ion‐conductive phase. c) Deconvolution of the FT‐IR spectra of pristine PSZ‐COF and the eutectic PSZ‐COF solid electrolyte for S═O vibrational modes. d) Li 1s XPS spectrum of the eutectic PSZ‐COF solid electrolyte. e) Deconvolution of the FT‐IR spectra of pristine PSZ‐COF containing LiFSI only and the eutectic PSZ‐COF solid electrolyte for coordinated and free FSI^−^ anions. f) Solid‐state ^7^Li NMR spectra of pure LiFSI and the eutectic PSZ‐COF solid electrolyte with a eutectic‐like ion‐conductive phase.

As depicted in **Figure** **3**a, the eutectic‐like ion‐conductive phase was created by making the Pyrrol‐FSI/zwitterion complex in the channels of pristine PSZ‐COF. The eutectic‐like ion‐conductive phase could accelerate the dissociation and migration of Li ions in the solid electrolyte. To confirm the eutectic‐like ion‐conductive phase in the eutectic PSZ‐COF solid electrolyte, several spectroscopic methods, including FT‐IR, ^7^Li‐NMR, and XPS, were employed. First, the vibrational modes of the sulfonate group in the zwitterion of pristine PSZ‐COF were investigated in the presence of Pyrrol‐FSI using FT‐IR spectroscopy. As shown in Figure 3b, the symmetric stretching mode of S═O in the sulfonate group showed a blue shift with peak broadening in the presence of both Pyrrol‐FSI and LiFSI (denoted Eutectic PSZ‐COF), compared with that in the pristine PSZ‐COF. As more clearly shown in the deconvoluted FT‐IR spectra (Figure 3c), the characteristic peak for the ionic complex between the sulfonate anion of PSZ‐COF and the pyrrolidinium cation of Pyrrol‐FSI strongly appeared at 1048 cm^−1^ in the eutectic PSZ‐COF solid electrolyte, whereas the zwitterionic SO_3_^−^ at 1035 cm^−1^ was dominant in the pristine PSZ‐COF.^[^ ^25^ ^]^ The chemical structure of the eutectic‐like ion‐conductive phase was further confirmed in the Li 1s XPS spectrum of the eutectic PSZ‐COF solid electrolyte (Figure 3d). The new characteristic peak for the Pyrrol‐FSI/zwitterionic SO_3_^−^ complex was clearly observed at 55.3 eV in addition to the peaks of LiSO_3_ (55.9 eV) and LiFSI (56.7 eV) in the XPS spectrum of the eutectic PSZ‐COF solid electrolyte. These experimental results obviously supported the formation of the eutectic‐like ion‐conductive phase in the solid electrolyte. The lower binding energy of Li ions in the Pyrrol‐FSI/zwitterionic SO_3_^−^ complex could allow their more facile dissociation in the eutectic PSZ‐COF solid electrolyte as compared to pure LiFSI salts.

The solvation environment of Li ions was identified to further verify the formation of the eutectic‐like ion‐conductive phase in the eutectic PSZ‐COF solid electrolyte. As shown in the FT‐IR spectrum of SO_2_ vibrational modes (Figure 3e), the SO_2_ proportion of a free FSI at 1357 cm^−1^ increased in the eutectic PSZ‐COF solid electrolyte compared with that in the pristine PSZ‐COF. In addition, the proportion of a coordinated FSI at 1380 cm^−1^ decreased in the eutectic PSZ‐COF solid electrolyte. This phenomenon was different from the previously reported tendency in the solvation of LiFSI in Pyrrol‐FSI,^[^ ^26^ ^]^ in which the coordinated FSI anions rather than free FSI were predominant. Therefore, a larger proportion of free FSI rather than coordinated FSI in the eutectic PSZ‐COF solid electrolyte can be an indicator of the formation of the eutectic‐like ion‐conductive phase depicted in Figure 3a. Subsequently, the coordination environment and mobility of Li ions were investigated by ^7^Li‐NMR, as shown in Figure 3f. As compared to pure LiFSI, the resonance peak of Li ions shifted toward downfield in the eutectic PSZ‐COF solid electrolyte, indicating a decrease in the electron density of Li cations.^[^ ^27^ ^]^ Besides, the full‐width at half maximum (FWHM) of the resonance peak at −0.1071 ppm was 114.22 Hz in the eutectic PSZ‐COF solid electrolyte, which was smaller than that in pure LiFSI. This unique chemical environment around Li ions supported the formation of the eutectic‐like ion‐conductive phase in the eutectic PSZ‐COF solid electrolyte. These results also indicated that the facile dissociation and rapid migration of Li ions could be realized in the eutectic PSZ‐COF solid electrolyte.^[^ ^28^ ^]^

### 2.3 Li‐Ion Transport and Electrochemical Properties of Eutectic PSZ‐COF Solid Electrolyte

**Figure 4:**
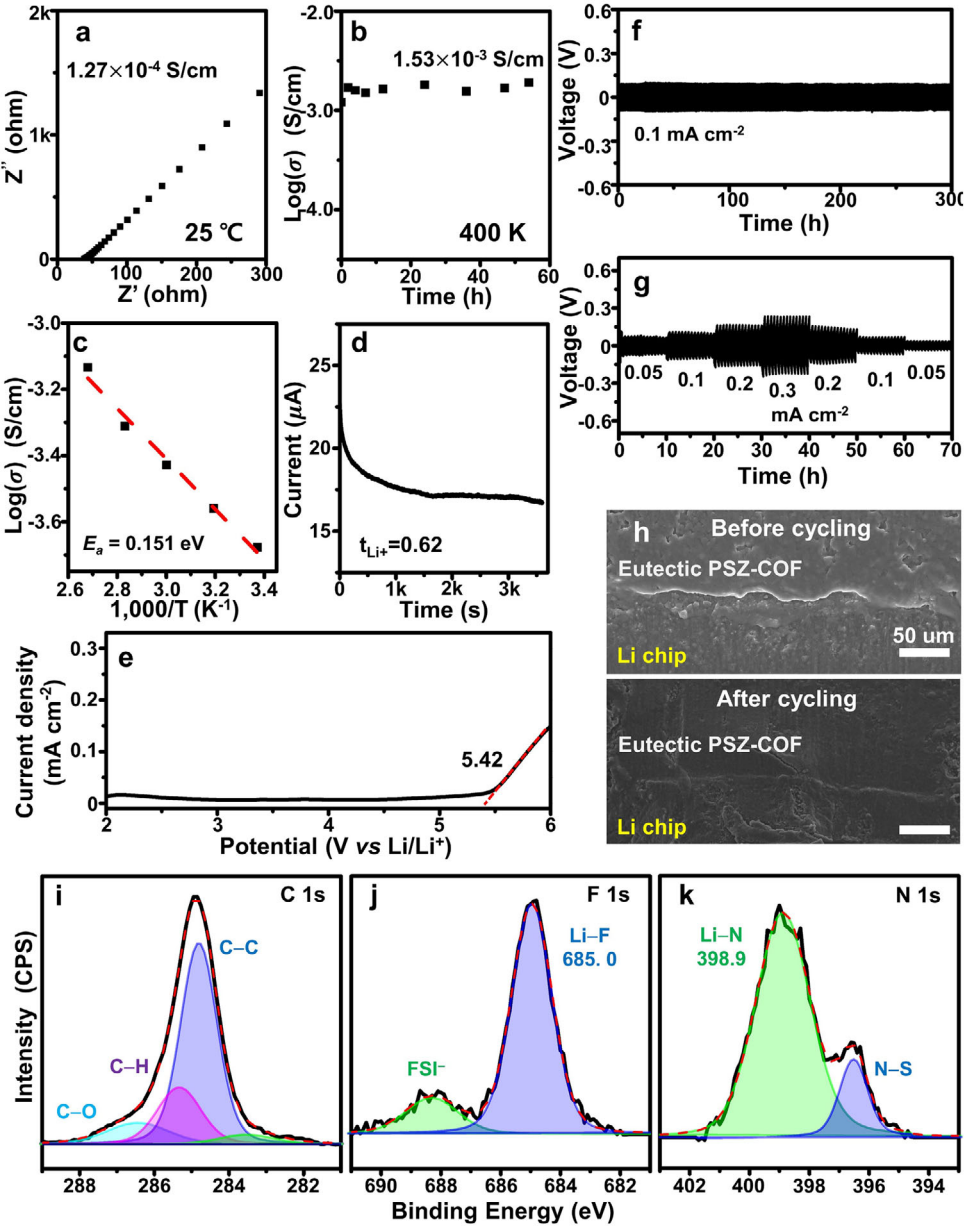
Li ion transport and electrochemical properties of eutectic PSZ‐COF solid electrolyte. a) Nyquist plot of a SS|eutectic PSZ‐COF|SS symmetric cell and the ionic conductivity of eutectic PSZ‐COF at room temperature. b) Thermal stability of the ionic conductivity of the eutectic PSZ‐COF solid electrolyte at a high temperature (400 K). c) Arrhenius plot of the ionic conductivities of the eutectic PSZ‐COF solid electrolyte at various temperatures. d) Chronoamperometry curve of a Li|eutectic PSZ‐COF|Li symmetric cell under 10 mV polarization at room temperature. e) Linear sweep voltammetry curve of a Li|eutectic PSZ‐COF|SS asymmetric cell at room temperature (scan rate 5 mV s^−1^). Li plating/stripping aspect of a Li|eutectic PSZ‐COF|Li symmetric cell at f) 0.1 mA cm^−2^ and g) at various current densities. h) Cross‐sectional SEM images of the eutectic PSZ‐COF solid electrolyte and Li metal interface before and after Li plating/stripping for 50 h. i) C 1s, j) F 1s, and k) N 1s XPS spectra of the SEI layer formed in the Li|eutectic PSZ‐COF|Li symmetric cell after Li plating/stripping for 50 h.

The eutectic PSZ‐COF solid electrolyte containing a Pyrrol‐FSI/zwitterion complex and LiFSI was made in pellet form using a pelletizer, which was then sandwiched with stainless steel (SS) to produce a SS|eutectic PSZ‐COF|SS symmetric cell. The room‐temperature ionic conductivity of the eutectic PSZ‐COF solid electrolyte was then measured by electrochemical impedance spectroscopy. As shown in **Figure** **4**a, the eutectic PSZ‐COF solid electrolyte exhibited a high ionic conductivity of 1.27 × 10^−4^ S cm^−1^ at room temperature. The eutectic PSZ‐COF solid electrolyte also exhibited superior thermal stability in ionic conductivity (1.53 mS cm^−1^) at an elevated temperature of 400 K for 50 h (Figure 4b). Furthermore, the temperature‐dependent ionic conductivity clearly suggested that the eutectic PSZ‐COF solid electrolyte displayed a low activation energy of 0.151 eV for Li‐ion transport (Figure 4c; Figure S13, Supporting Information). It was noteworthy that the pristine PSZ‐COF without a eutectic‐like Pyrrol‐FSI/zwitterion complex showed a lower room‐temperature ionic conductivity of 7.28 × 10^−5^ S cm^−1^ and a higher activation energy of 0.378 eV (Figures S14–S16, Supporting Information). These experimental results distinctly revealed that the eutectic‐like Pyrrol‐FSI/zwitterion complex phase of the eutectic PSZ‐COF solid electrolyte could accelerate the dissociation and migration of Li ions at room temperature.

The Li‐ion transference number (t_Li+_) of the eutectic PSZ‐COF solid electrolyte at room temperature was also measured in the Li|eutectic PSZ‐COF|Li symmetric cell using the Bruce‐Vincent method. The chronoamperometry curve of the Li symmetric cell showed that the eutectic PSZ‐COF solid electrolyte exhibited a very high t_Li+_ of 0.62 at room temperature (Figure 4d; Figure S17, Supporting Information), which was comparable with that of liquid electrolytes. The greater room‐temperature t_Li+_ could be attributed to the fact that the flexible eutectic‐like conductive phase allowed fast transport of Li ions in the eutectic PSZ‐COF solid electrolyte while the cationic pyridinium site could trap the FSI anions. For the pristine PSZ‐COF bearing LiFSI only, the value of t_Li+_ (0.47) appeared to be much smaller in Figures S18 and S19 (Supporting Information). Subsequently, the electrochemical stability of the eutectic PSZ‐COF solid electrolyte was found by the LSV curve of the SS|eutectic PSZ‐COF|Li asymmetric cell at a scan rate of 5 mV s^−1^. As shown in Figure 4e, the eutectic PSZ‐COF solid electrolyte exhibited superior oxidative resistance by 5.42 V, allowing its utilization in high‐voltage Li metal batteries.

Next, the Li plating and stripping performance of the eutectic PSZ‐COF solid electrolyte at room temperature was confirmed in the Li symmetric cell. As shown in Figure 4f, a stable and narrow overpotential without short‐circuit was observed in the eutectic PSZ‐COF solid electrolyte during successive Li plating/stripping cycles for 300 h at 0.1 mA cm^−2^. Furthermore, the Li plating/stripping process remained stable and reversible at various current densities from 0.05 to 0.3 mA cm^2^ (Figure 4g). This outstanding Li plating/stripping performance in the Li symmetric cell revealed that the eutectic PSZ‐COF solid electrolyte was very compatible with a reactive Li metal anode. Then, the suppression of Li dendrites by the eutectic PSZ‐COF solid electrolyte was confirmed by scanning electron microscopy (SEM). As shown in the cross‐sectional SEM images of Li anodes (Figure 4h), the smooth and compact interface between the eutectic PSZ‐COF solid electrolyte and the Li metal was retained even after successive cycles for 50 h. No Li dendrites and voids were created on the Li metal anode. Furthermore, the solid electrolyte interface (SEI) layer formed in the Li plating/stripping cycles was identified by XPS. The C 1s XPS spectrum in Figure 4i showed that no characteristic peaks for carbonate groups were observed,^[^ ^29^ ^]^ while the very intense peak for Li−F clearly appeared at 685.0 eV (Figure 4j).^[^ ^30^ ^]^ In addition, the strong peak for Li−N at 398.9 eV was observed in the N 1s XPS spectrum (Figure 4k).^[^ ^31^ ^]^ These analytical results revealed that the eutectic PSZ‐COF solid electrolyte also drove the formation of an ion‐conductive LiF‐ and Li_3_N‐rich SEI layer on a Li metal anode.

### 2.4 Li‐Ion Diffusion Dynamics in Eutectic PSZ‐COF Solid Electrolyte

**Figure 5:**
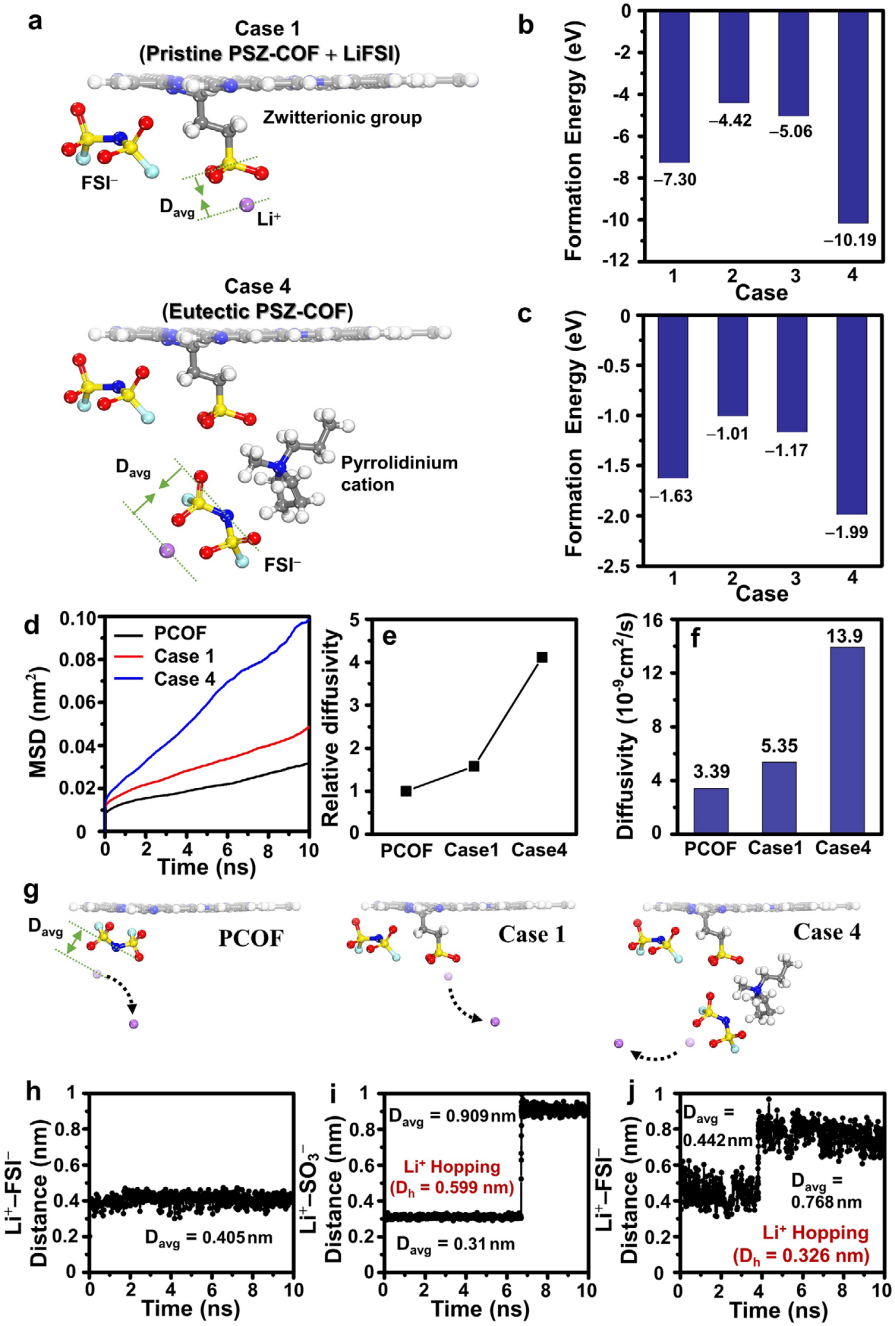
Li‐ion diffusion dynamics in eutectic PSZ‐COF solid electrolyte. a) Schematic illustration for the plausible structures of the pristine PSZ‐COF complex (Case 1) and the eutectic PSZ‐COF complex (Case 4). Formation energies of four different PSZ‐COF complexes calculated by b) DFT, and c) DFT‐COSMO considering the experimental dielectric environment. Case 1 consists of the zwitterionic pyridinium group of pristine PSZ‐COF and a LiFSI salt. Case 2 includes a pyrrolidinium cation instead of Li^+^ in Case 1. Case 3 involves an additional FSI‐ anion in Case 2. Case 4 includes a eutectic‐like ion‐conductive phase formed by addition of Pyrrolidinium‐FSI into Case 1. d) Mean squared displacement (MSD) as a function of time for PCOF, Case 1, and Case 4 at 398 K. e) Relative diffusivity and f) Calculated diffusion coefficients for PCOF, Case 1, and Case 4. g) Schematic illustrations of Li‐ion dissociation for PCOF, Case 1, and Case 4. Time‐evolved distance between the Li cation and the FSI anion or the zwitterionic group, showing the Li‐ion diffusion mechanism, and the average distance (D_avg_) and the hopping distance (D_h_) for h) PCOF, i) Case 1, and j) Case 4.

DFT calculations were performed to gain deeper insight into the formation of Li hopping sites in the eutectic PSZ‐COF solid electrolyte. The plausible structures induced by the combination of cations (pyridinium, pyrrolidinium, Li^+^) and anions (FSI^−^ and SO_3_^−^) were categorized into four distinct cases, depicted in **Figures** **5**a and S20 (Supporting Information). Based on the systematic design of the possible structures, both DFT and DFT‐COSMO calculations were carried out to evaluate the formation energies of all the cases (Figure 5b,c). The simulation results indicated that the eutectic‐like ion‐conductive phase corresponding to Case 4 exhibited the lowest formation energy in both DFT and DFT‐COSMO calculations. This suggested that the eutectic‐like ion‐conductive phase was most favorable in the eutectic PSZ‐COF solid electrolyte as Li‐ion hopping sites. In Case 1 (not containing Pyrrol‐FSI), the negatively charged sulfonate of the zwitterionic group stabilized a Li ion, while the positively charged pyridinium ring on the COF plane interacted with a FSI anion. These interactions induced a balanced charge distribution that can reduce the electrostatic potential and enhance the system stability, leading to the lower formation energy. As a further development, the incorporation of Pyrrol‐FSI in the zwitterionic group (Case 4) promoted the formation of a eutectic‐like configuration, as characterized by regularly alternating the cations and the anions through electrostatic attraction. This specific arrangement could facilitate optimal charge distribution throughout the solid electrolyte system, effectively mitigating local charge imbalances. As a result, this ordered ionic configuration can create the most favorable electrostatic environment, leading to the lowest formation energy observed in the eutectic PSZ‐COF solid electrolyte. These findings provided insight into the stable formation of a eutectic‐like ion‐conductive pathway for Li‐ion transport in the eutectic PSZ‐COF solid electrolyte, regardless of the experimental dielectric environment.

MD simulations along with DFT calculations were further conducted to elucidate the enhanced Li‐ion conductivity of the eutectic PSZ‐COF solid electrolyte. To this end, the dissociation energy (E_d_) and diffusivity of Li ions were analyzed within the favorable stacking structures of PCOF and PSZ‐COF. To investigate the effect of Pyrrol‐FSI on migration of Li ions in the solid electrolytes, DFT and DFT‐COSMO simulations were first performed to compare the dissociation energies of Li ions in three different structures: PCOF, pristine PSZ‐COF, and eutectic PSZ‐COF. As depicted in Figure 5a, Case 4 corresponded to the eutectic PSZ‐COF solid electrolyte, while Case 1 represented the pristine PSZ‐COF bearing LiFSI. A key factor for improving Li‐ion conductivity is the effective dissociation of LiFSI salts, which are strongly bound by Coulombic interactions, increasing the number of mobile ions. As shown in Figure S21a (Supporting Information), the relative dissociation energy (E_d_) of LiFSI salts was obtained for three structures: PCOF, Case 1, and Case 4. The dissociation energy of an isolated LiFSI pair was calculated to be 5.99 eV,^[^ ^32^ ^]^ which was then used as a reference for normalization to obtain the relative E_d_. Among them, Case 4 for the eutectic PSZ‐COF solid electrolyte had the lowest E_d_ value of 0.83, followed by Case 1 of pristine PSZ‐COF (0.96) and PCOF (0.97). Similarly, the eutectic PSZ‐COF solid electrolyte (Case 4) also exhibited a lower dissociation energy in the experimental dielectric environment (DFT‐COSMO) compared to the other two structures (Figure S21b, Supporting Information). The lower value in E_d_ implied easier dissociation of Li ions, showing that the eutectic PSZ‐COF solid electrolyte could more easily promote dissociation of LiFSI salts than PCOF and pristine PSZ‐COF. The Case 4 structure corresponding to the eutectic PSZ‐COF solid electrolyte displayed a well‐distributed charge due to the ordered arrangement of Pyrrol‐FSI. Even though the overall stability of the system was improved, this led to relative weakening in the Coulombic interaction between a Li cation and a FSI anion, compared to that in PCOF and pristine PSZ‐COF. As a result, Li ions could diffuse via a hopping mechanism,^[^ ^33^ ^]^ driven by the combination of the favorable eutectic‐like ion‐conductive configuration and the lowest dissociation energy of Li ions in the eutectic PSZ‐COF solid electrolyte.

The mean square displacement (MSD) analysis using MD simulations found that the eutectic PSZ‐COF solid electrolyte in Case 4 had a diffusivity of 13.9 × 10^−9^ cm^2^ s^−1^, which was higher than that of PCOF (3.39 × 10^−9^ cm^2^ s^−1^) and Case 1 of pristine PSZ‐COF (5.35 × 10^−9^ cm^2^ s^−1^), as shown in Figure 5d–f. These values were in good agreement with the experimental results. To understand the difference in the diffusivity of Li ions in three different structures (PCOF, pristine PSZ‐COF, and eutectic PSZ‐COF), the time‐evolved distance between the Li ion and its interacting anion was further simulated (Figure 5g–j). Figure 5h showed that a Li ion did not dissociate from an FSI^−^ anion in the PCOF structure due to their strong Coulombic interaction, as the time‐evolved average distance (D_avg_) remained at 0.405 nm over 10 ns. In contrast, hopping motions were observed in both Case 1 and Case 4. The time‐evolved average distance between the Li ion and the sulfonate anion of the zwitterionic group in Case 1 increased from 0.310 to 0.909 nm at 6.7 ns, with a hopping distance (D_h_) of 0.599 nm (Figure 5i). Meanwhile, in Case 4 of the eutectic PSZ‐COF, the distance between the Li ion and the FSI anion shifted from 0.442 to 0.768 nm at 3.8 ns, with a hopping distance of 0.326 nm (Figure 5j). These results indicated that the hopping motion of Li ions could occur more rapidly in Case 4 of eutectic PSZ‐COF than in Case 1 of pristine PSZ‐COF due to the lower energetic barriers originating from the shorter hopping distance and lower dissociation energy of Li ions. Specifically, the favorable formation of the eutectic‐like ion‐conductive pathway created by incorporation of Pyrrol‐FSI in the eutectic PSZ‐COF solid electrolyte can significantly shorten the hopping distance, making Li ions easier to migrate. Consequently, the eutectic PSZ‐COF solid electrolyte exhibited superior ionic conductivity and larger Li‐ion transference number at room temperature. These theoretical interpretations emphasized that the eutectic PSZ‐COF solid electrolyte could provide an effective route to improve the room‐temperature ionic conductivity and Li‐ion transference number of COF‐based solid electrolytes.

### 2.5 Performance of All‐Solid‐State Li Metal Batteries

**Figure 6:**
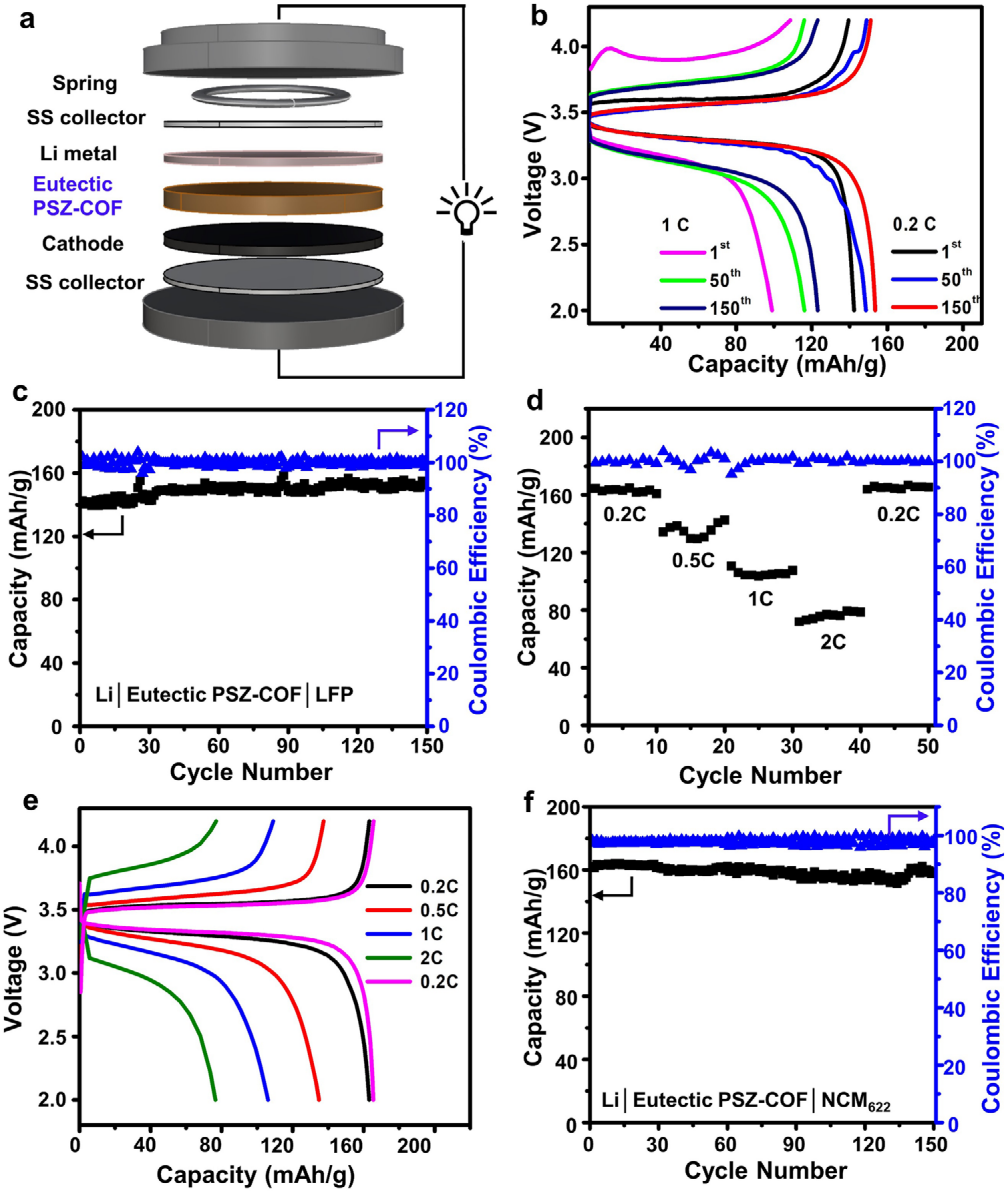
Performance of all‐solid‐state Li metal batteries with eutectic PSZ‐COF solid electrolyte. a) Schematic illustration of all‐solid‐state Li metal full cell with the eutectic PSZ‐COF solid electrolyte (Li|eutectic PSZ‐COF|LFP or NCM_622_). b) Long‐term charge/discharge voltage profiles of the Li|eutectic PSZ‐COF|LFP full cells at 0.2C and 1C for 150^th^ cycle at room temperature. c) Long‐term cycling performance of the Li|eutectic PSZ‐COF|LFP full cell at 0.2C at rt. d) C‐rate‐dependent capacity and Coulombic efficiency of the Li|eutectic PSZ‐COF|LFP full cell at rt. e) Charge/discharge voltage profiles of the Li|eutectic PSZ‐COF|LFP full cell at various C‐rates. f) Long‐term cycling performance of the Li|eutectic PSZ‐COF|NCM_622_ full cell at 0.2C at room temperature.

To evaluate the eutectic PSZ‐COF solid electrolyte in Li metal batteries, all‐solid‐state Li metal full cells were assembled with a LFP or NCM_622_ cathode as shown in **Figure** **6**a. The long‐term cyclic performance of the Li|eutectic PSZ‐COF|LFP cell was first examined at 0.2 and 1 C (Figure 6b). The all‐solid‐state Li metal batteries with the eutectic PSZ‐COF solid electrolyte retained a high capacity of 153.3 mAh g^−1^ at 0.2C and 123.1 mAh g^−1^ at 1 C, respectively, during 150 cycles. Figure 6c also demonstrated that the eutectic PSZ‐COF solid electrolyte enabled the all‐solid‐state Li metal battery to exhibit a 100% retention of specific capacity (153.6 mAh g^−1^ at 0.2C) and an outstanding Coulombic efficiency of 99.9% after 150 cycles. Besides, the all‐solid‐state Li metal battery showed stable cycle performance at a higher C‐rate (1C) in Figure S22 (Supporting Information), in which the initial discharge capacity of 98.9 mAh g^−1^ increased to 123.1 mAh g^−1^ at 150th cycle. This increase in the discharge capacity might be attributed to the formation of the stable and ion‐conductive SEI layer observed in Figure 4h‐‐k as the cycle number increased. These results clearly revealed that the eutectic PSZ‐COF solid electrolyte was very compatible with Li metal batteries of high capacity. Subsequently, the rate performance of the Li|eutectic PSZ‐COF|LFP full cell was investigated. As shown in Figure 6d, the all‐solid‐state Li metal cell exhibited stable and reversible discharge capacities with a high Coulombic efficiency of 99.9% at various C‐rates from 0.2, 0.5, 1, to 2C, indicating that the eutectic PSZ‐COF solid electrolyte remained constant in its ionic conductivity and electrochemical stability. The corresponding charge/discharge profile of the full cell showed specific capacities of 163.1, 134.9, 106.1, 76.2, and 165.5 mAh g^−1^ at 0.2, 0.5, 1, 2, and 0.2 C, respectively (Figure 6e).

Given the wide electrochemical stability window of the eutectic PSZ‐COF solid electrolyte, an all‐solid‐state Li metal full cell with a NCM_622_ cathode (Li|eutectic PSZ‐COF|NCM_622_) was also assembled, and its long‐term cyclic performance was investigated. As shown in Figure 6f, the NCM_622_ full cell with the eutectic PSZ‐COF solid electrolyte displayed a stable discharge capacity of 158.8 mAh g^−1^ with a high Coulombic efficiency of 97.7% for 150 cycles at 0.2 C. Furthermore, the all‐solid‐state NCM_622_ full cell showed a stable and reversible rate performance at 0.2, 0.5, 1, 2, and 0.2 C with a high capacity of 188.0, 165.9, 129.9, 47.2, and 182.3 mAh g^−1^, respectively (Figures S23 and S24, Supporting Information). All these battery performances clearly revealed that the eutectic PSZ‐COF solid electrolyte with superior room‐temperature ionic conductivity and electrochemical stability could provide a new route for high energy‐density Li metal batteries with improved safety.

The post‐mortem analysis of all‐solid‐state Li metal batteries was further carried out after 150 cycles.^[^ ^34^ ^]^ As shown in Figure S25 (Supporting Information), the charge transfer resistance (R_ct_) of the Li|eutectic PSZ‐COF|LFP full cell decreased after 150 cycles because of the formation of a LiF/Li_3_N‐rich SEI layer identified by XPS.^[^ ^35^ ^]^ The Li|eutectic PSZ‐COF|NCM_622_ full cell,r, showed a slight increase in the charge transfer resistance after 150 cycles (Figure S26, Supporting Information). This might be attributed to the lower stability of NCM_622_ at higher operating voltages.^[^ ^36^ ^]^ As shown in the FT‐IR spectra of the eutectic PSZ‐COF solid electrolyte (Figures S27 and S28, Supporting Information), the characteristic vibrational modes for its pyridinium, pyridine, and sulfonate groups were still observed at 1687, 1638, 1214, and 1054 cm^−1^, respectively, after 150 cycles of the all‐solid‐state full cells with LFP or NCM_622_. Furthermore, the cross‐sectional SEM images of the full cells showed the smooth interfaces between the electrode and the eutectic PSZ‐COF solid electrolyte after 150 cycles (Figure S29, Supporting Information).

## 3 Conclusion

The eutectic PSZ‐COF solid electrolyte was simply prepared by making a complex of Pyrrol‐FSI and LiFSI with the zwitterionic group of pristine PSZ‐COF in the ordered channel. The formation of the eutectic‐like ion‐conductive phase in the eutectic PSZ‐COF solid electrolyte was confirmed by various spectroscopic methods. The as‐prepared eutectic PSZ‐COF solid electrolyte exhibited a high room‐temperature ionic conductivity of 1.27 × 10^−4^ S cm^−1^ and a large room‐temperature Li‐ion transference number of 0.62. Furthermore, the eutectic PSZ‐COF solid electrolyte had superior thermal stability and wide electrochemical stability. DFT and MD simulations found that a time‐evolved average distance and a hopping distance were significantly reduced in the eutectic PSZ‐COF solid electrolyte, leading to the rapid migration of Li ions. The eutectic PSZ‐COF solid electrolyte successfully suppressed the formation of Li dendrites and also drove the formation of a uniform ion‐conductive SEI layer on a Li anode during long‐term cycles. Finally, the all‐solid‐state lithium metal battery, utilizing a eutectic PSZ‐COF solid electrolyte and an LFP cathode with a high active material loading of 2.0 mg cm^−^^2^, demonstrated a high initial specific capacity of 153.3 mAh g^−1^, a high Coulombic efficiency of 99.9%, and capacity retention of 100% during 150 cycles at room temperature. As an NCM_622_ cathode was used in the all‐solid‐state Li metal battery, the battery performance was also outstanding, showing a specific capacity of 162.1 mAh g^−1^, a Coulombic efficiency of 97.7%, and capacity retention of 98.3%. This eutectic PSZ‐COF solid electrolyte can offer an effective way to design high‐performance organic solid electrolytes and will be applied in various forms of all‐solid‐state Li batteries.

## Conflict of Interest

The authors declare no conflict of interest.

## Supporting information



Supporting Information

## Data Availability

The data that support the findings of this study are available from the corresponding author upon reasonable request.
