# Supplementary material for: Eutectic‐Like Ion‐Conductive Phase‐Incorporated Zwitterionic Covalent Organic Framework Solid Electrolyte for All‐Solid‐State Li Metal Batteries
Source: Adv Sci (Weinh). 2025 Jun 25;12(33):e05530. doi: 10.1002/advs.202505530 (PMC12412492; doi:10.1002/advs.202505530)
Supplement: Supplementary file 1 — Supporting Information [file ADVS-12-e05530-s001.pdf]

ADVANCED SCIENCE
Open Access

Supporting Information

for *Adv. Sci.*, DOI 10.1002/advs.202505530

Eutectic-Like Ion-Conductive Phase-Incorporated Zwitterionic Covalent Organic Framework Solid Electrolyte for All-Solid-State Li Metal Batteries

Jaewoo Lee, Jae-Hoon Shin, Sungpyo Hong, Jun-Hyeong Lee, Dae-Hui Jeong, Hongwon Kim, Yong Hui Kim, Sang Uck Lee and Jong-Ho Kim**

Copyright WILEY-VCH Verlag GmbH & Co. KGaA, 69469 Weinheim, Germany, 2018.

Supporting Information

Eutectic-like Ion-Conductive Phase-incorporated Zwitterionic Covalent Organic Framework Solid Electrolyte for All-Solid-State Li Metal Batteries

Jaewoo Lee[+], Jae-Hoon Shin[+], Sungpyo Hong, Jun-Hyeong Lee, Dae-Hui Jeong, Hongwon Kim, Yong Hui Kim, Sang Uck Lee, and Jong-Ho Kim**

J. Lee[+], J.H. Shin[+], J.H. Lee, D.H. Jeong, H. Kim, J.H. Kim*
Department of Materials Science and Chemical Engineering, Hanyang University, Ansan 15588, Republic of Korea
*Email: kjh75@hanyang.ac.kr
[+]These authors contributed equally to this work

S. Hong, Y.H. Kim, S.U. Lee*
School of Chemical Engineering, Sungkyunkwan University, Suwon 16149, Republic of Korea
*Email: suleechem@skku.edu

Materials: 1,3,5-Triformylbenzene (≥98%), LiFSI (>98%) , 1,3-propanesultone (>99%), mesitylene (≥97%), Pyrrol-FSI (>98%), *N*-methyl pyrrolidone (NMP, anhydrous, ≥99.5%), and lithium nickel cobalt manganese oxide ($LiNi_{0.6}Co_{0.2}Mn_{0.2}O_2$, ≥99.9%, NCM_{622}) were purchased from TCI Chemicals (Japan). 2,6-Diaminopyridine (≥98%), poly(vinylidene difluoride) (PVDF, M_W = 534,000), *N*,*N*-diisopropylethylamine (DIEA, ≥99%), and 1,4-dioxane (anhydrous, ≥ 99.8%) were purchased from Sigma-Aldrich (USA). Acetone, tetrahydrofuran, and acetic acid (≥99.7%) were purchased from Daejung Chemicals (Korea). LFP, cell cases, Li metal chips, spring, and stainless steel spacers were purchased from MTI Korea. Conductive carbon (Super P) was purchased form Alfa Aesar (USA).

Instruments: Physical morphology and surface structure of COF and solid electrolytes were characterized by high-resolution TEM (HR-TEM, JEM-F200, JEOL) and scanning electron microscopy (SEM, S-4800, Hitachi). Pore size distribution and BET surface area were measured using the Brunauer-Emmett-Teller method (BET, 3-Flex, Micromeritics). Crystallinity was confirmed by X-ray diffraction (XRD, D/max 2500, Rigaku). Elemental compositions were identified by elemental analyzer (2400 Series II CHNS/O Analyzer, PerkinElmer). Chemical structures were characterized using an FT-IR spectrometer (IS10, Thermo Fisher Scientific), NMR (AVANCE II + 400, Bruker), and XPS (AXIS-NOVA and Ultra DLD, Kratos Analytical). A centrifuge (CombiS14R, Hanil Scientific) was used for isolation of PCOF and PSZ-COF. For electrochemical analysis, a potentiostat (VersaSTAT 3, Ametek) and battery cycler (WBCS3000, WonAtech) were used.

Synthesis of PCOF: Pyridine-rich COF (PCOF) was synthesized by Schiff base reaction of 2,6-diaminopyridine and 1,3,5-triformylbenzene. A 450 mg portion of 1,3,5-triformylbenzene

and 455 mg of 2,6-diaminopyridine were dissolved in 60 mL of 1,4-dioxane and mesitylene (1:1 vol%). After sonication for 10 min, 6 mL of 6M acetic acid was added to the mixture solution. Then, the resulting mixture was stirred at 120°C for 72 h. After cooling the solution to room temperature, it was centrifuged at 12000 rpm for 10 min, and the product PCOF was washed with THF several times using a centrifuge. PCOF was obtained after drying it under vacuum for 24 h.

***Synthesis of pristine PSZ-COF*:** A 300 mg portion of PCOF was dispersed in 30 mL of NMP, in which 288 μL of DIEA was added and stirred for 5 min at room temperature to neutralize it. After addition of 270 mL ethyl acetate into the solution, PCOF was precipitated and filtrated. The neutralized PCOF was washed with water and ethyl acetate three times each. Then, 200 mg of the neutralized PCOF was dispersed in 40 mL of NMP, in which 450 mg of 1,3-propanesulltone was added. The resulting mixture solution was then stirred at 70°C for 24 h. After cooling the solution to room temperature immediately, the product PSZ-COF was precipitated by adding 360 mL ethyl acetate. The pristine PSZ-COF powder was collected by vacuum filtration, followed by drying it under vacuum for 24 h.

***Preparation of Eutectic PSZ-COF Solid Electrolyte*:** First, 100 mg of LiFSI were mixed with 73 μL Pyrrol-FSI, which was then ground on a mortar. Then, 100 mg of pristine PSZ-COF was added into the mixture of LiFSI and Pyrrol-FSI, which was further ground for 30 min. The mixture was placed in a pelletizer and gently pressed to obtain a solid electrolyte pellet. After drying it at 80°C for 24 h, the thickness of the eutectic PSZ-COF solid electrolyte was measured. All procedures proceeded in a glovebox.

The pristine PSZ-COF solid electrolyte not containing a eutectic-like ion-conductive phase was also prepared as follows. A 100 mg of LiFSI and 100 mg of pristine PSZ-COF were mixed with 60 μL of NMP, which was then ground for 30 min. Then, the mixture was placed in a pelletizer and gently pressed. The obtained pellet was dried at 80°C for 24 h.

***Measurement of ionic conductivity*:** Ionic conductivities were measured with a stainless steel (SS) spacer|eutectic PSZ-COF|SS symmetric cell (CR2032-coin cell) using electrochemical impedance spectroscopy (EIS) at an applied amplitude of 10 mV and a frequency range from 10^6 to 1 Hz. Ionic conductivity (σ) was calculated by the following equation where l represents the thickness of a solid electrolyte, A is the surface area of a solid electrolyte, and R_b is the bulk resistance of a cell. R_b was measured between the semicircle and the charge transfer linear polarization curve.

$$\sigma = \frac{l}{A \times R_b} \text{ [S cm}^{-1}\text{]}$$

***Measurement of electrochemical stability window*:** The electrochemical stability window of the eutectic PSZ-COF solid electrolyte was obtained by linear sweep voltammetry (LSV) curves of a SS|eutectic PSZ-COF|Li asymmetric cell under a sweep rate of 5 mV/s with a voltage range from 1.5 V to 6 V.

***Measurement of Li-ion transference number*:** The Li-ion transference number (t_{Li+}) of the eutectic PSZ-COF solid electrolyte at room temperature was measured using the Bruce-Vincent method[1]. A Li|eutectic PSZ-COF|Li symmetric cell was analyzed by EIS. The alternating current (AC) impedance of the symmetric cell was measured before and after direct current

(DC) polarization. The t_{Li+} of the eutectic PSZ-COF solid electrolyte was calculated by the following equation.

$$t_{Li+} = \frac{I_{ss}\cdot(\Delta V - I_0 R_0)}{I_0\cdot(\Delta V - I_{ss} R_{ss})}$$

where I_{ss} and I_0 represent a steady-state current and an initial current, respectively. R_{ss} and R_0 are interfacial resistances before and after polarization, respectively, and ΔV is an applied voltage of 10 mV.

***Li plating/stripping test and battery performance*:** The Li plating and stripping behavior of the eutectic PSZ-COF solid electrolyte was analyzed using a Li|eutectic PSZ-COF|Li symmetric cell under 0.1 mA/cm^2 at room temperature. The Li|eutectic PSZ-COF|Li symmetric cell was analyzed for 300 h, and each cycle was performed for 40 min.

For measurement of the performance of all-solid-state Li metal batteries having a eutectic PSZ-COF solid electrolyte, a LFP or NCM_{622} cathode was first custom-made. For preparation of a LFP cathode, LFP, Super P, and PVDF were mixed at a mass ratio of 8:1:1 in NMP, followed by stirring it for 24 h to make the cathode slurry. Then, the slurry was cast on aluminum foil and then dried at 80°C for 24 h. The foil was punched to obtain small discs with a diameter of 1.5 cm in which the active cathode materials were loaded in ~2.0 mg/cm^2. The specific capacity was calculated by multiplying the area capacity (mAh/cm^2) by the area of the aluminum foil, which was then divided by the mass of the active material. A NCM_{622} cathode was prepared using the same procedure as the LFP cathode except for the use of NCM_{622} instead of LFP.

The long-term cycle performance and charge/discharge profile of all-solid-state Li metal batteries were investigated in a voltage range from 2.0 V to 4.2 V for LFP full cells or 2.8 V to 4.3 V for NCM_{622} full cells. For measurement of the rate-performance of all-solid-state Li metal batteries, C-rates were varied from 0.2 to 2 C in a voltage range from 2.0 V to 4.2 V for LFP

full cells or 3.0 V to 4.5 V for NCM_{622} full cells. The long-term cycle performance of the batteries was tested at 0.2 and 1 C (ca. 0.31 mA/cm^2) for 150 cycles (LFP full cells) and 100 cycles (NCM_{622} full cells), respectively.

Electrochemical impedance spectroscopy (EIS) of the Li|eutectic PSZ-COF|LFP (or NCM_{622}) full cell (CR2032-coin cell) was conducted at an applied amplitude of 10 mV and a frequency range from 10^6 to 1 Hz.

***Computational Simulation Details*:** All computational simulations were conducted using the Materials Studio 2024 software package [2]. For geometric optimization and MD simulations for PCOF and pristine PSZ-COF, the Forcite module was employed [3]. The simulation parameters for both COF structures, including those governing bonded and nonbonded interactions, were derived from the condensed-phase optimized molecular potentials for atomistic simulation studies III (COMPASSIII) force field [4]. An atomic charge of +1.0 e was assigned to each Li^+ and pyrrolidinium cation, with an equal but opposite charge assigned to each FSI^-, ensuring the electrical neutrality of the simulation box. The simulation parameters were consistently implemented in both DFT and MD calculations.

To determine the most energetically stable configurations of PCOF and pristine PSZ-COF, we analyzed the possible stacking structures of AA, AB1, and AB2, which are shown in Figure S1. After geometry optimization, PCOF and PSZ-COF adopted the AA and AB1 stacking modes, respectively as depicted in Figure S2. In these calculations, lattice constants and internal atomic positions were fully optimized until the residual forces were below 0.001 kcal/mol/Å, the energy change was less than $2 * 10^{-5}$ kcal/mol, and the atomic displacement was smaller than $1 * 10^{-6}$nm. Electrostatic interactions were treated using the Particle-Particle Particle-Mesh (PPPM) method. Van der Waals interactions were computed using an atom-based scheme. Following structural optimization, the diffusion of Li ions within these structures was

investigated using a 10 ns MD simulation at 398 K with a time step of 1 fs. The simulation was performed in an NVT ensemble employing a Nose–Hoover thermostat[5]. Understanding the mechanism underlying Li-ion diffusion at room temperature is challenging, as it requires simulation times beyond the nanosecond scale. To gain insight into the role of the eutectic-like phase involving Pyrrol-FSI in facilitating the Li-ion transport in the eutectic PSZ-COF solid electrolyte, simulations were conducted at a higher temperature of 398 K. This approach aimed to enhance the understanding of Li-ion diffusion kinetics under conditions that accelerate the diffusion process.

To compare the simulated structures with the experimental X-ray crystal structures of PCOF and pristine PSZ-COF, their powder XRD patterns were calculated using the Reflex module [6]. Copper (Cu) was selected as the X-ray source, with a 2-theta scanning range of 3° to 50° and a step size of 0.001°.

The formation energy of possible PSZ-COF electrolyte structures and the dissociation energy of Li ions were computed via DFT calculations using in the DMol3 module [7]. Both energy terms were calculated based on isolated moieties and salt species (e.g., pyrrolidinium cation, Li^+, FSI^-), without considering periodic boundary conditions. Specifically, the Perdew-Burke-Ernzerhof (PBE) functional within the generalized gradient approximation (GGA) and the DNP basis set were employed for electron density calculations. To ensure computational convergence during geometry optimization, the criteria included an energy convergence tolerance of 10^{-5} Hartree, a maximum force limit of 0.002 Hartree/Å, and a displacement threshold of 0.0005 nm with all-electron treatment applied to all atoms. Additionally, the conductor-like screening model (COSMO) [8] was utilized to represent the experimental dielectric environment using a dielectric constant of 13.41[9] for Pyrrol-FSI. The overall computational scheme and key parameters are summarized in Table S2.

References

[1] J. Evans, C. A. Vincent, P. G. Bruce, *Polymer* **1987**, *28*, 2324-2328.
[2] Materials studio 2024.
[3] R. G. U. Shankar, S.K. sethi, A. Verma, *Forcefields for Atomistic-Scale Simulations: Materials and Applications*, **2022**.
[4] a)X. D. Li, S. Q. Feng, F. Guo, X. Y. Liu, J. X. Yu, Z. W. Hou, *Rsc Adv* **2016**, *6*, 21517-21525; b)H. Sun, *J Phys Chem B* **1998**, *102*, 7338-7364.
[5] N. Shuichi, *Constant Temperature Molecular Dynamics Methods, Progress of Theoretical Physics Supplement, Vol. 103*, **1991**.
[6] S. J. Lyle, R. W. Flaig, K. E. Cordova, O. M. Yaghi, *J Chem Educ* **2018**, *95*, 1512-1519.
[7] a)B. Delley, *J Chem Phys* **2000**, *113*, 7756-7764; b)B. Delley, *J Chem Phys* **1990**, *92*, 508-517.
[8] a)B. Delley, *Mol Simulat* **2006**, *32*, 117-123; b)H. Kwon, H. Kim, J. Hwang, W. Oh, Y. Roh, D. Shin, H. T. Kim, *Nat Energy* **2024**, *9*, 57-69.
[9] A. Rybinska-Fryca, A. Sosnowska, T. Puzyn, *J Mol Liq* **2018**, *260*, 57-64.

Supporting Figures

Table S1. Elemental composition of pristine PSZ-COF measured by elemental analyzer

Element	Elemental analysis (wt%)
C	56.11
H	5.21
N	16.27
S	6.25

Table S2. Computational schemes and key parameters used in molecular dynamics (MD) and density functional theory (DFT) simulations

**Molecular dynamics (MD)**		
**Scheme**		**Condition and Parameter**
Forcefield		Condensed-phase optimized molecular potentials for atomistic simulation studies III (COMPASSIII)
Convergence tolerance	Energy<br>Force<br>Displacement	2×10^{-5} kcal/mol<br>0.001 kcal/mol/Å<br>1×10^{-6} nm
Summation method	Electrostatic<br>van der Waals	Particle-Particle Particle-Mesh method (PPPM)<br>Atom based
Dynamics	Ensemble<br>Time step<br>Thermostat	NVT<br>1 fs<br>Nose

**Density functional theory (DFT)**		
**Electronic Hamiltonian**	**Scheme**	**Condition and Parameter**
Exchange-correlation energy	Generalized-gradient approximation (GGA)	Perdew-Burke-Ernzerhof (PBE)
Core treatment	All electron	
Basis set	DNP	Basis file 4.4
SCF	Tolerance<br>Multipolar expansion<br>Charge density mixing	1×10^{-6} eV/atom<br>Hexadecapole<br>Charge 0.2, DIIS 6
Orbital cutoff	Global	0.37 nm

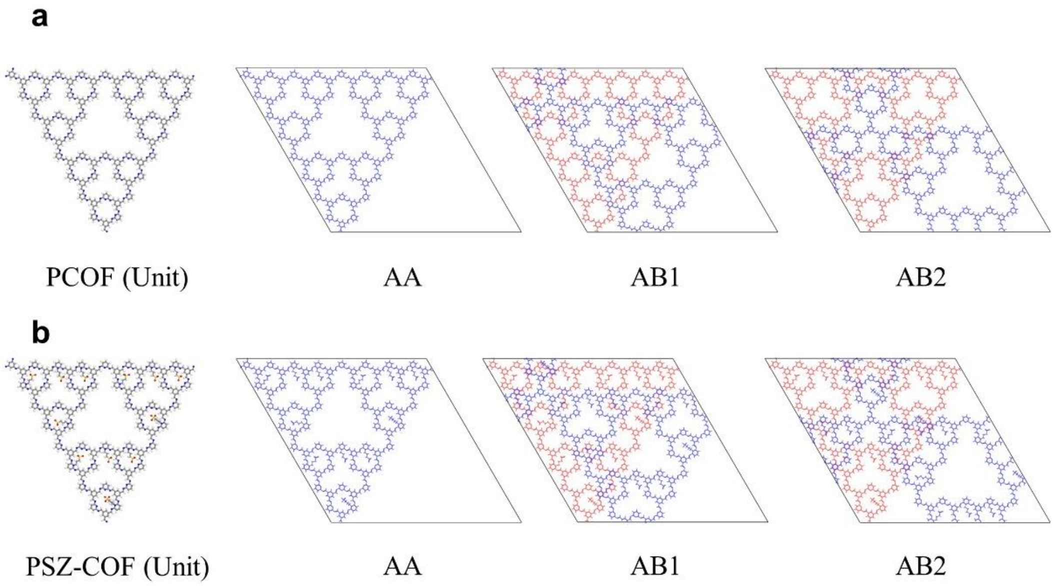

Figure S1. Stacking structures (AA, AB1 and AB2) of **a)** PCOF and **b)** Pristine PSZ-COF.

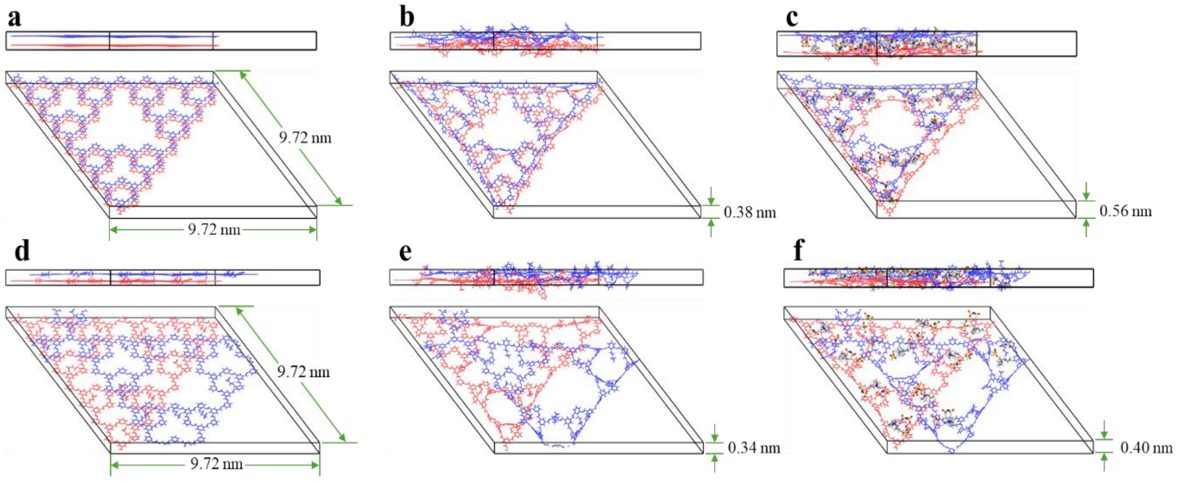

Figure S2. Representative molecular dynamics (MD) simulations of **a)-c)** PCOF and **d)-f)** PSZ-COF. Initial configurations prior to geometry optimization (a & d); PCOF adopts an AA stacking mode, while PSZ-COF exhibits AB1 stacking configuration. Optimized structures with refined inter-layer spacing (b & e). Final configurations after incorporation of LiFSI salts and pyrrolidinium cations, followed by further structural optimization (c & f).

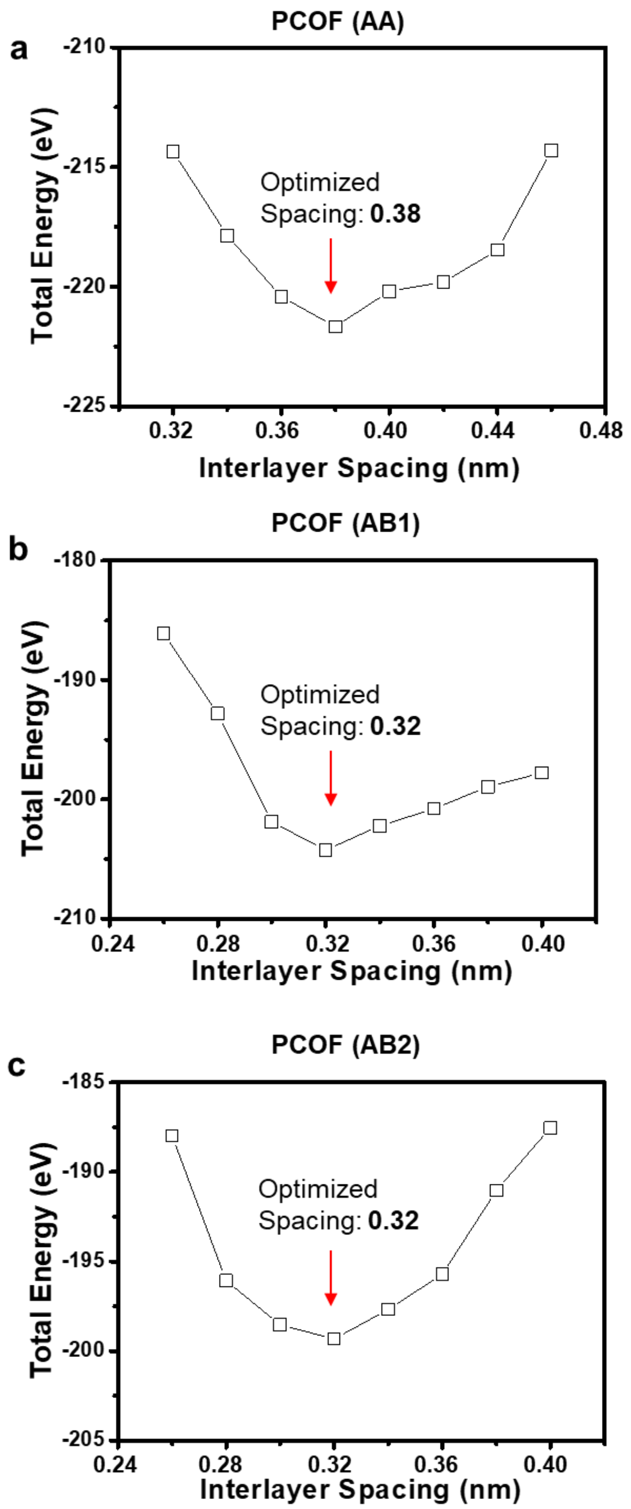

Figure S3. Total energy versus interlayer spacing for various configurations of PCOF. The graphs display the optimized interlayer spacing values for **a)** AA, **b)** AB1, and **c)** AB2 configurations of PCOF with the corresponding total energy minimum indicated by a red arrow.

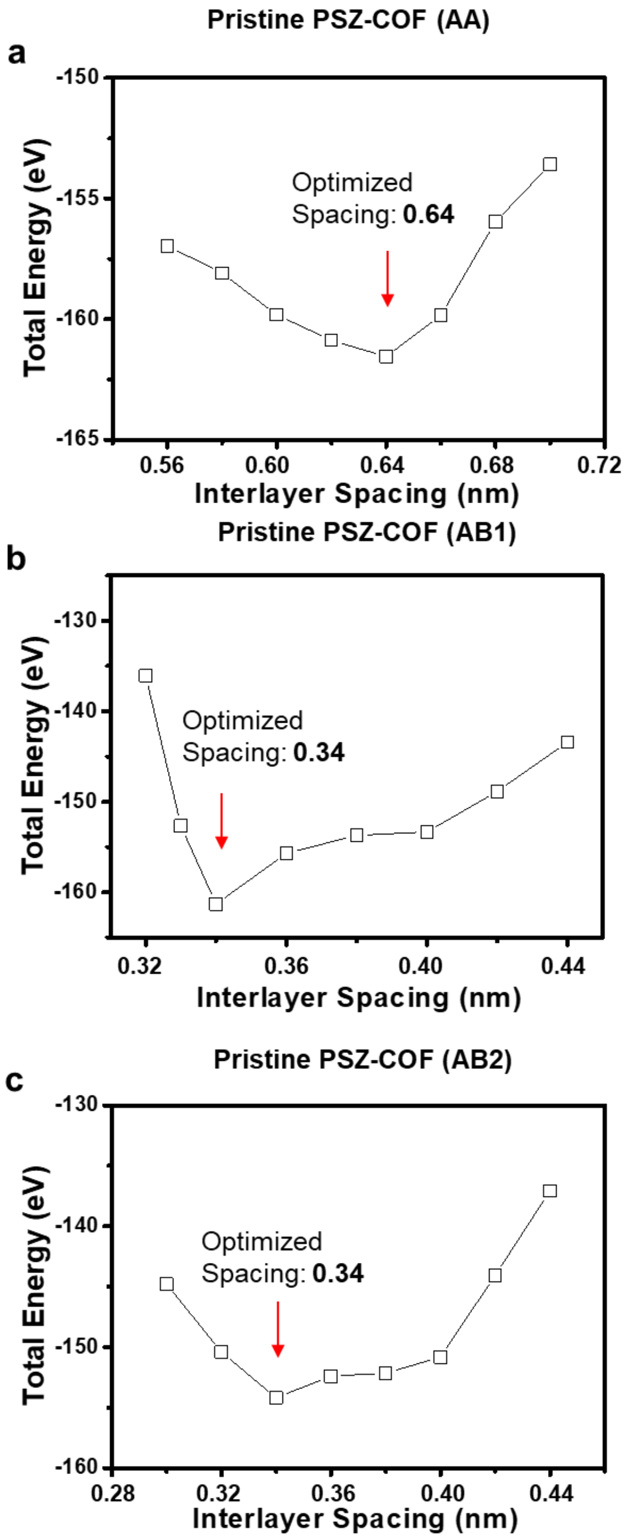

Figure S4. Total energy versus interlayer spacing for various configurations of pristine PSZ-COF. The graphs display the optimized interlayer spacing values for **a)** AA, **b)** AB1, and **c)** AB2 configurations of PSZ-COF with the corresponding total energy minimum indicated by a red arrow.

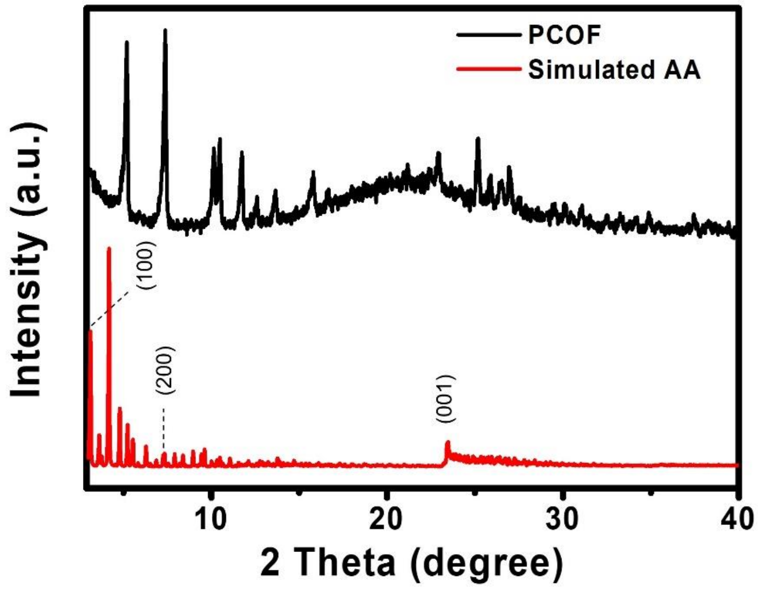

Figure S5. Measured and simulated XRD patterns of PCOF.

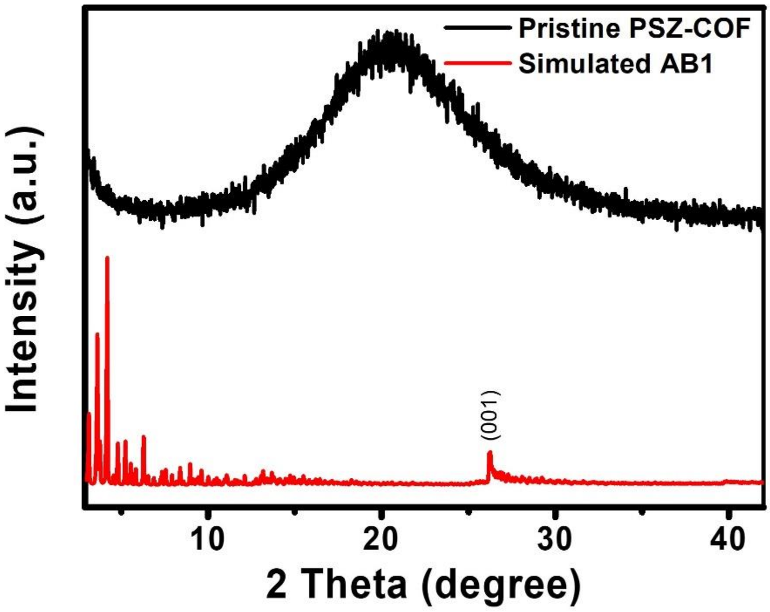

Figure S6. Measured and simulated XRD patterns of pristine PSZ-COF.

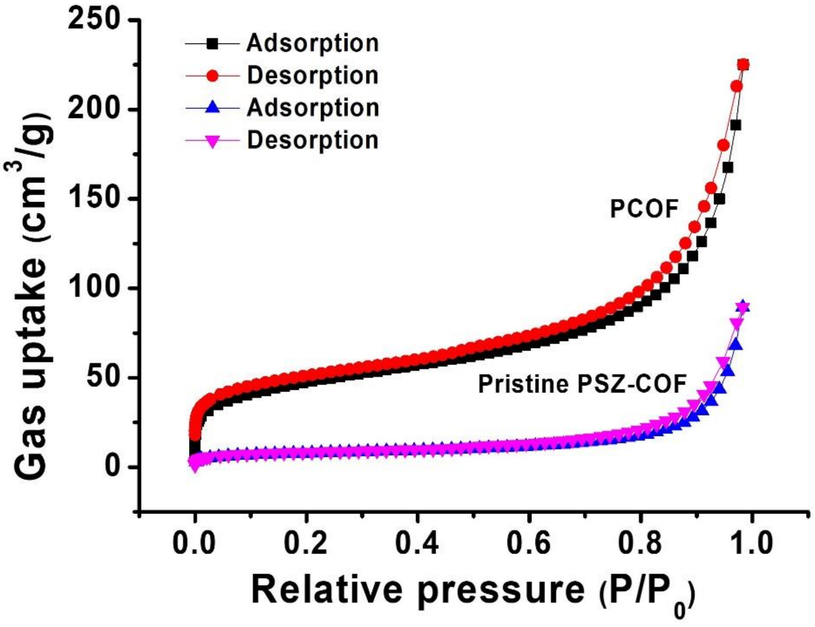

Figure S7. N_2 adsorption and desorption isotherms of PCOF and pristine PSZ-COF.

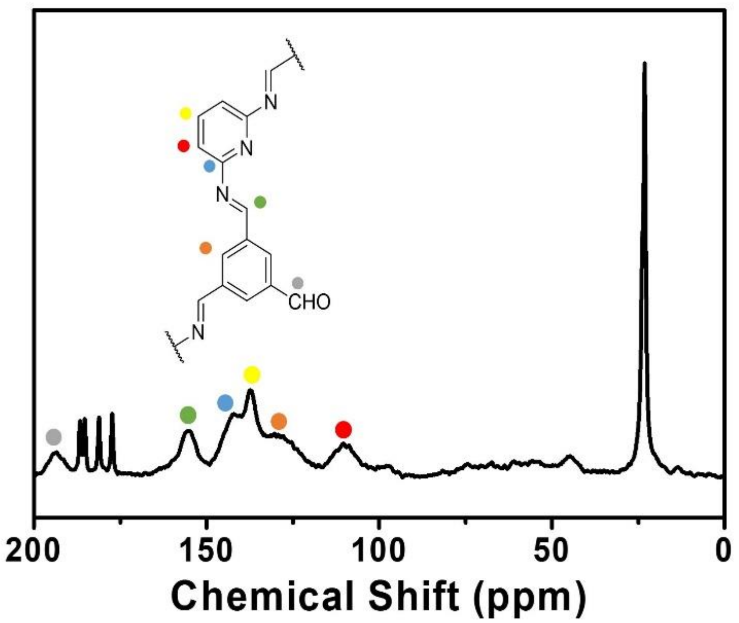

Figure S8. ^{13}C-NMR spectrum of PCOF.

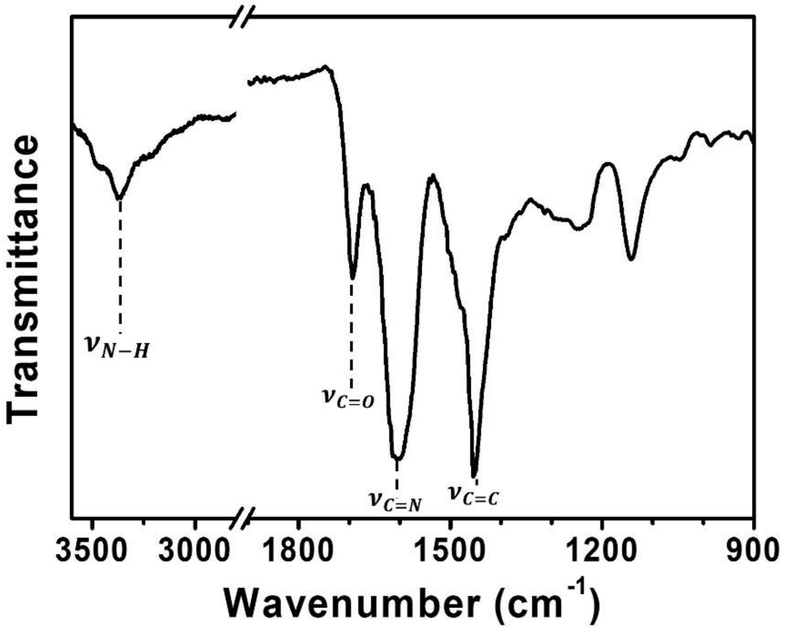

Figure S9. FT-IR spectrum of PCOF.

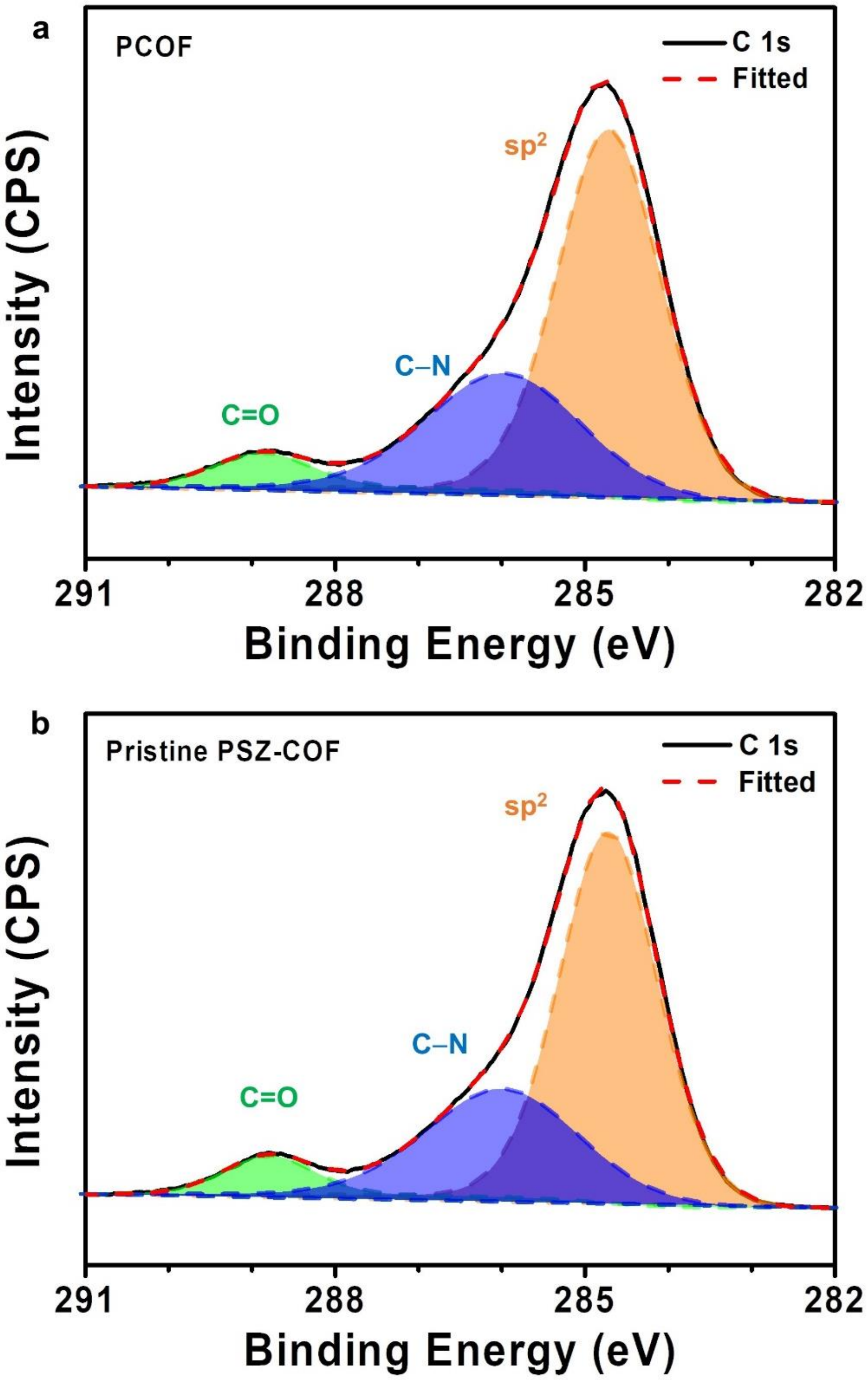

Figure S10. C 1s XPS spectra of **a)** PCOF and **b)** Pristine PSZ-COF.

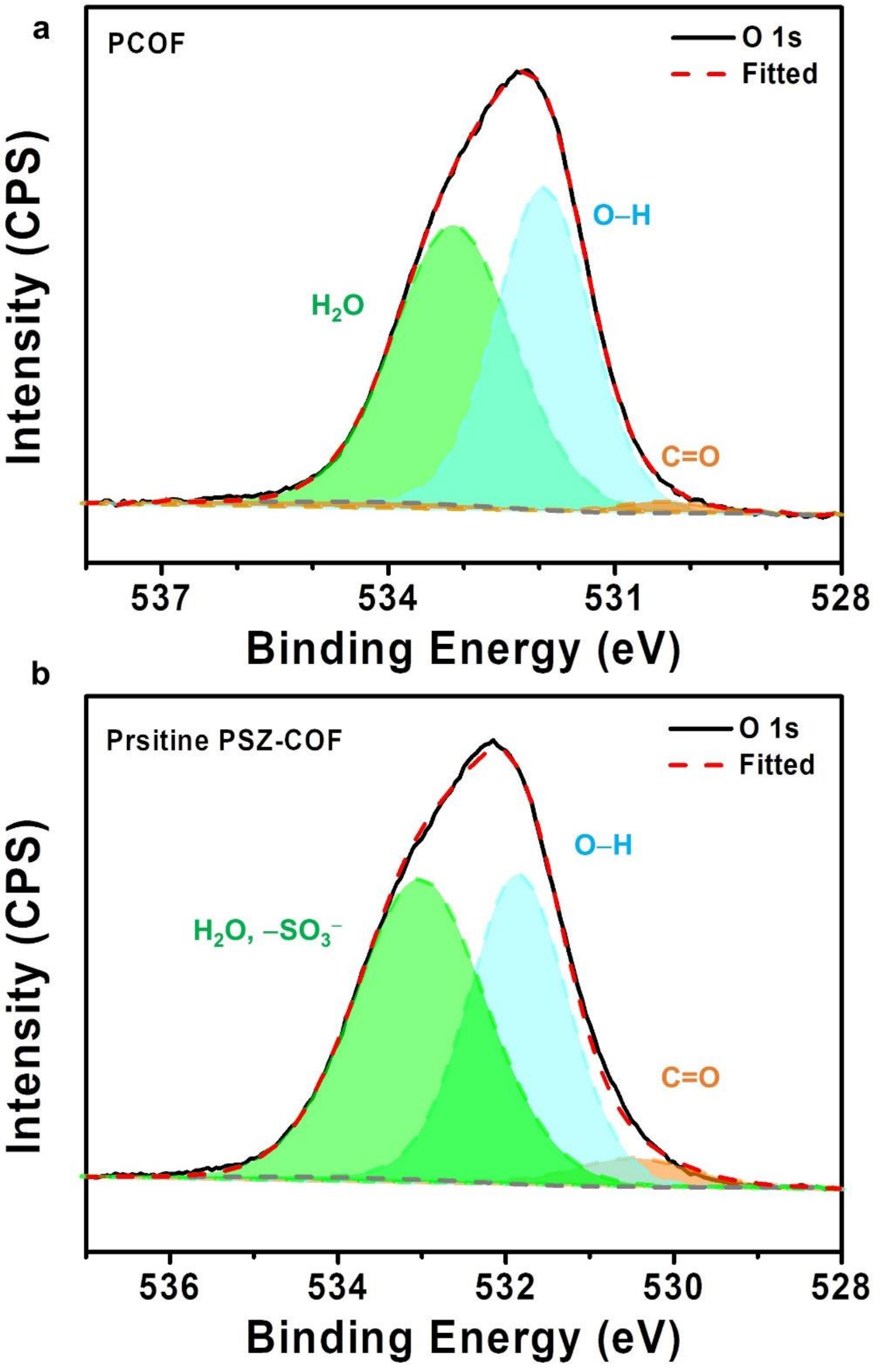

Figure S11. O 1s XPS spectra of **a)** PCOF and **b)** Pristine PSZ-COF.

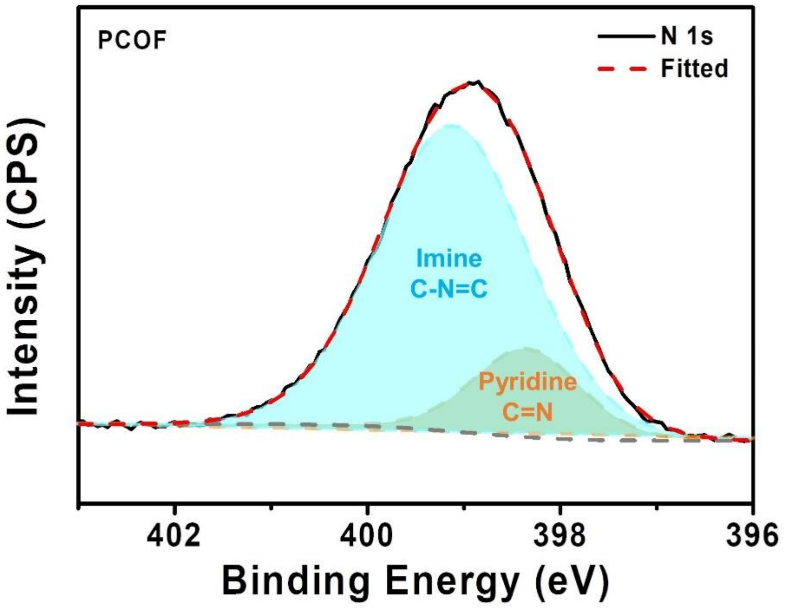

Figure S12. N 1s XPS spectrum of PCOF.

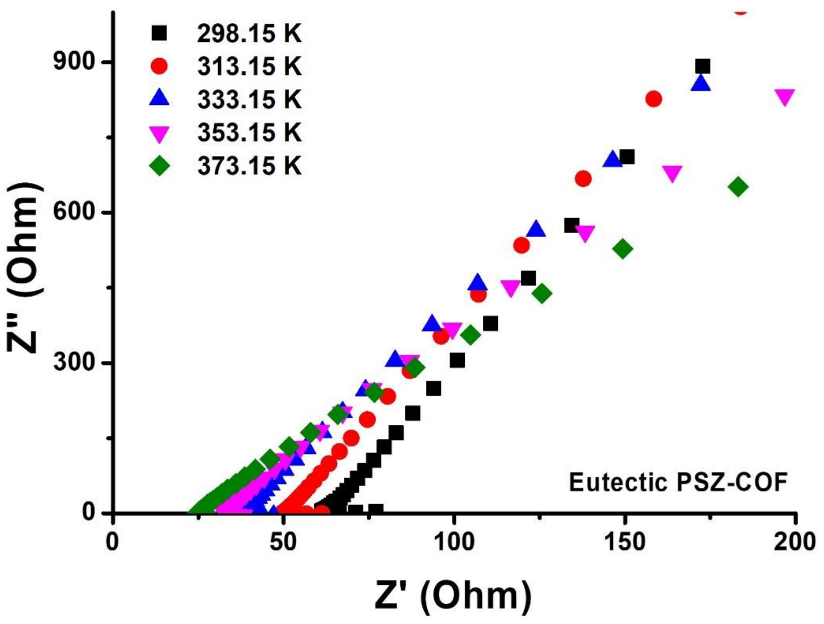

Figure S13. Nyquist plots of eutectic PSZ-COF solid electrolyte at various temperatures.

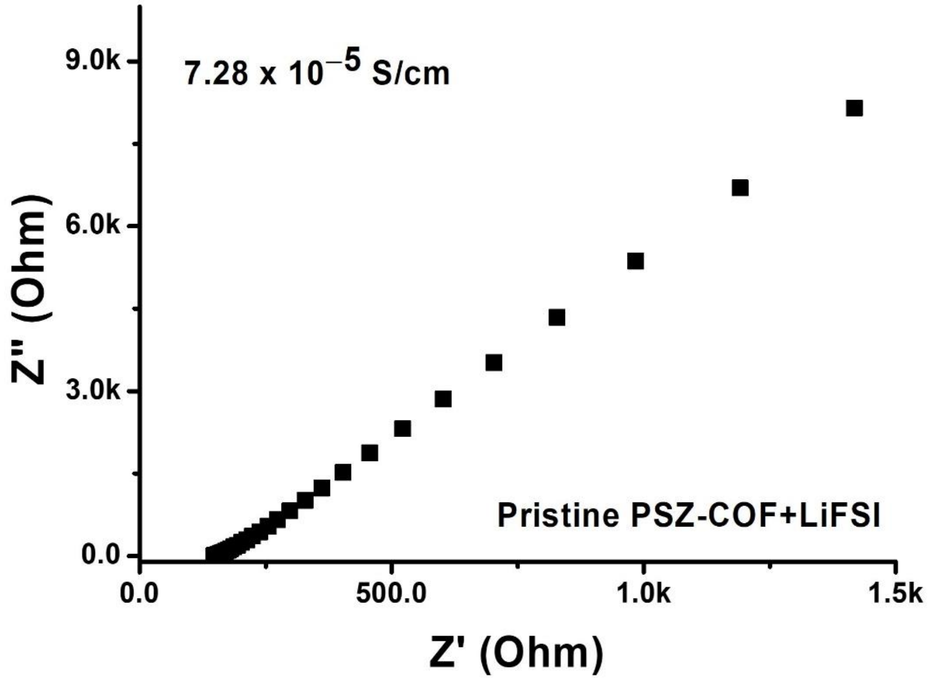

Figure S14. Ionic conductivity of pristine PSZ-COF bearing LiFSI at room-temperature.

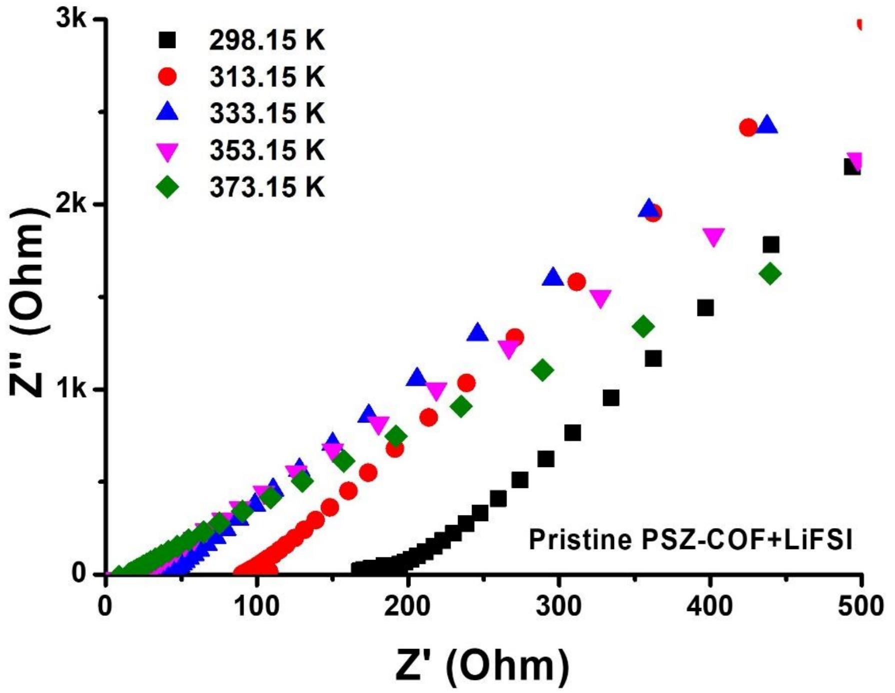

Figure S15. Nyquist plots of pristine PSZ-COF bearing LiFSI at various temperatures.

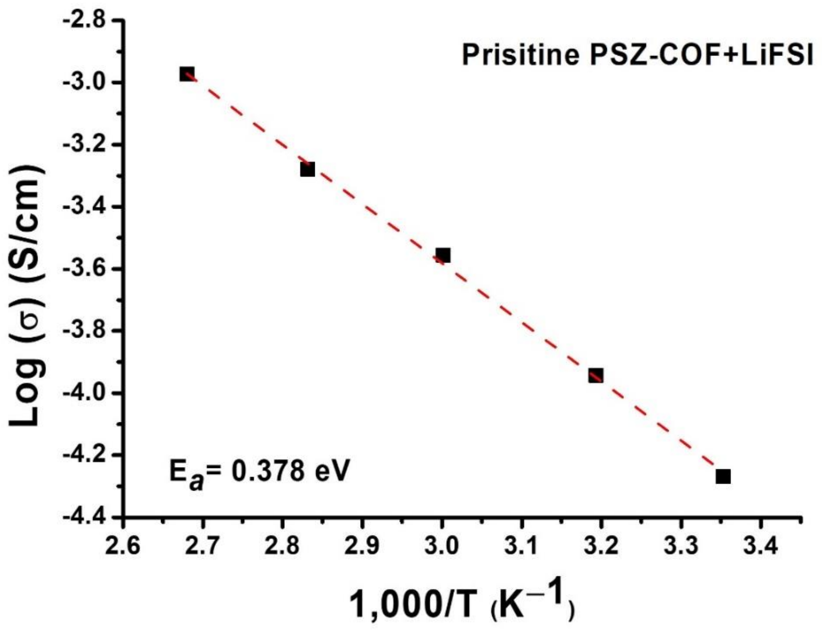

Figure S16. Ionic conductivities of pristine PSZ-COF solid electrolyte bearing LiFSI as function of temperature and its calculated activation energy.

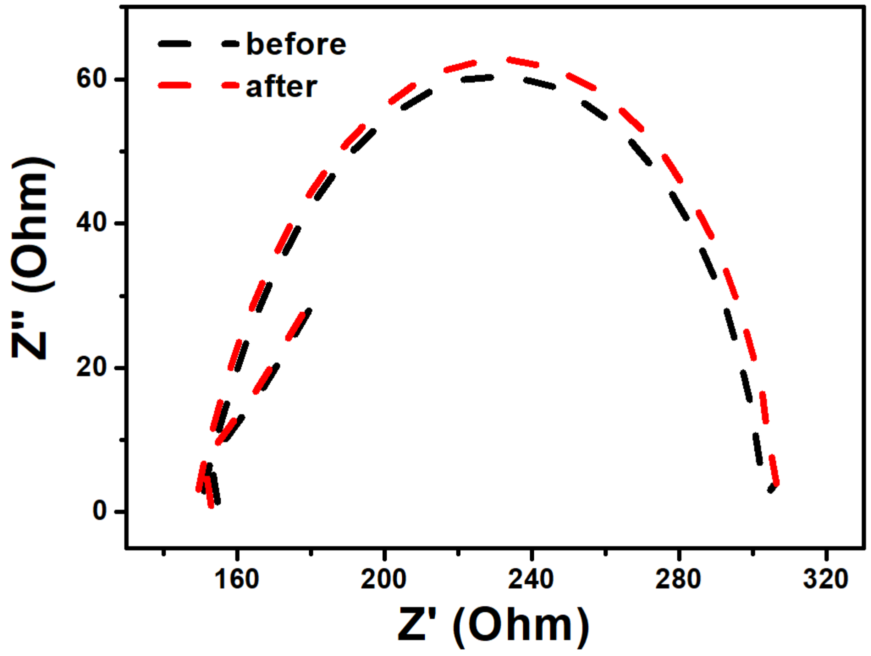

Figure S17. EIS curves of eutectic PSZ-COF solid electrolyte before and after polarization at 10 mV.

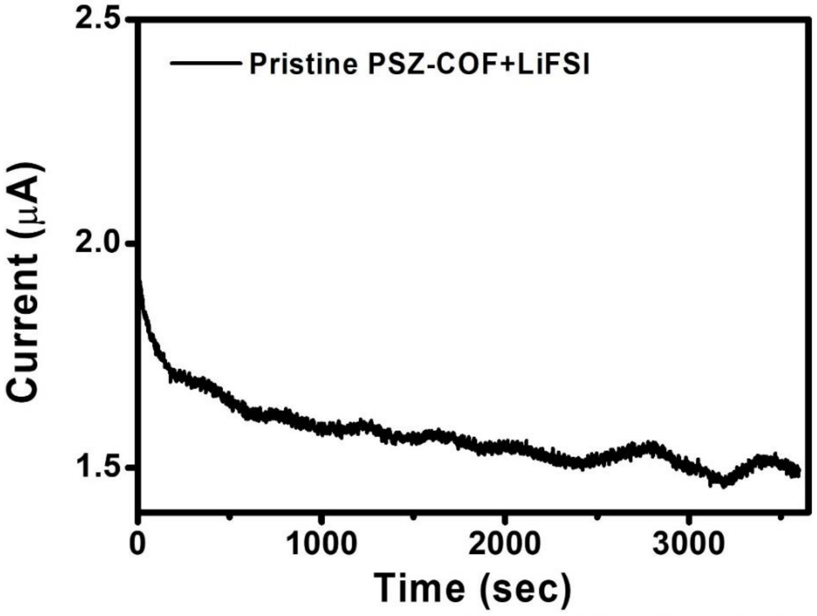

Figure S18. Chronoamperometry curve of the pristine PSZ-COF bearing LiFSI only under 10 mV at room temperature.

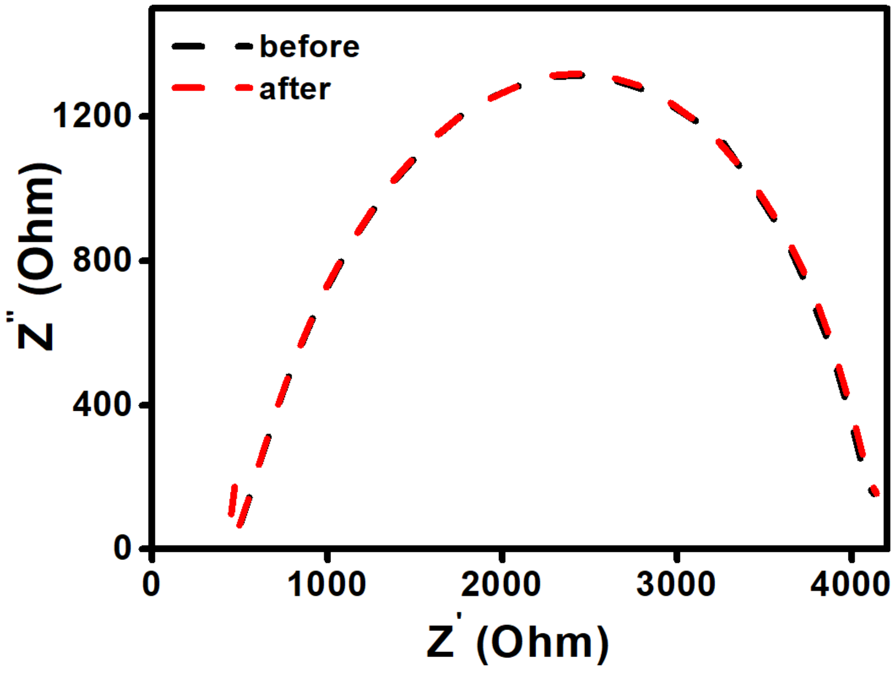

Figure S19. EIS curves of the pristine PSZ-COF bearing LiFSI before and after the polarization at 10 mV.

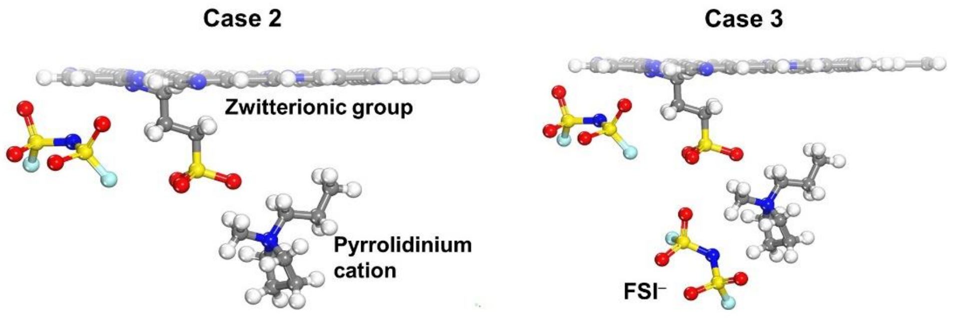

Figure S20. Schematic illustrations for the possible structures of PSZ-COF complexes. Case 2 contains a pyrrolidinium cation instead of a Li^+ ion in Case 1. Case 3 is negatively charged due to the addition of additional FSI^- ion to Case 2.

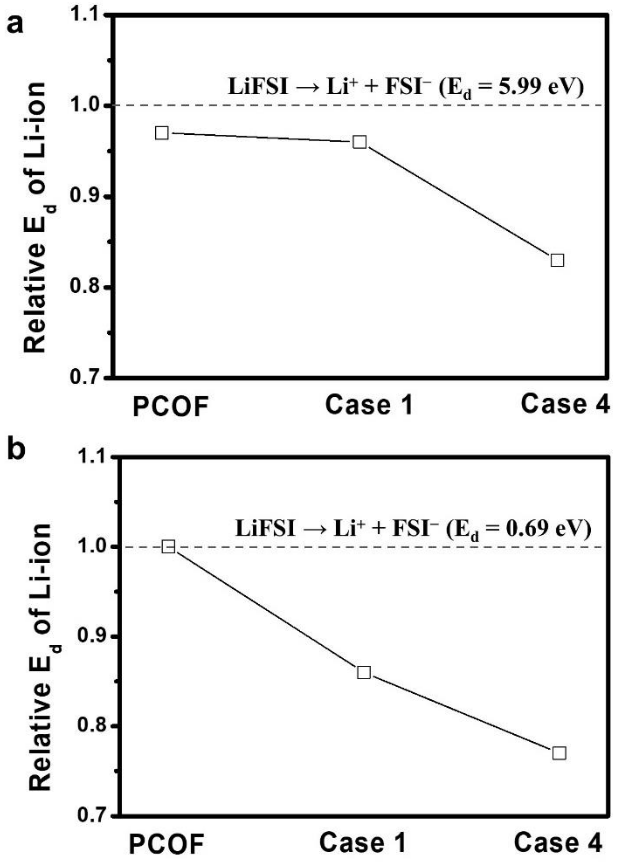

Figure S21. Relative dissociation energies (E_d) of Li ions calculated by **a)** DFT and **b)** DFT-COSMO. The dashed line indicates the dissociation energy of the LiFSI ion pair without any other interacting species.

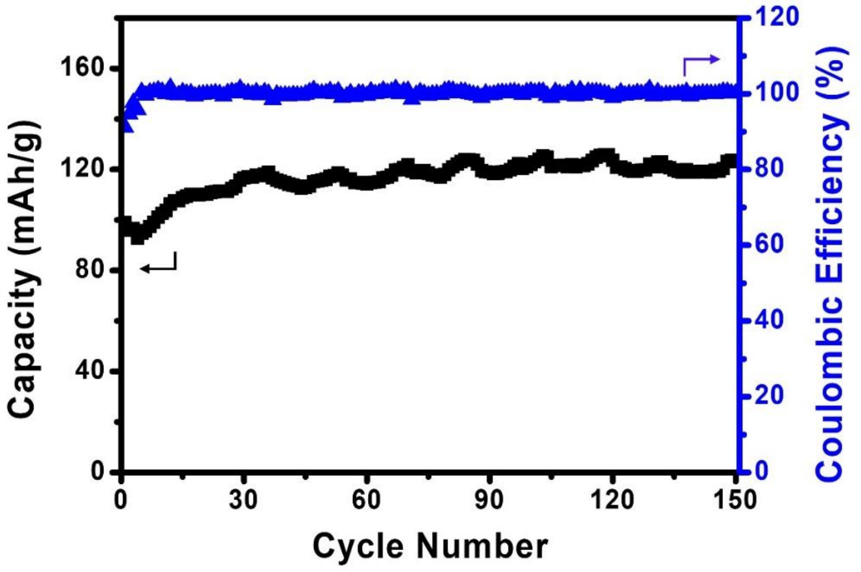

Figure S22. Cycle performance of the Li|eutectic PSZ-COF|LFP full cell at 1 C.

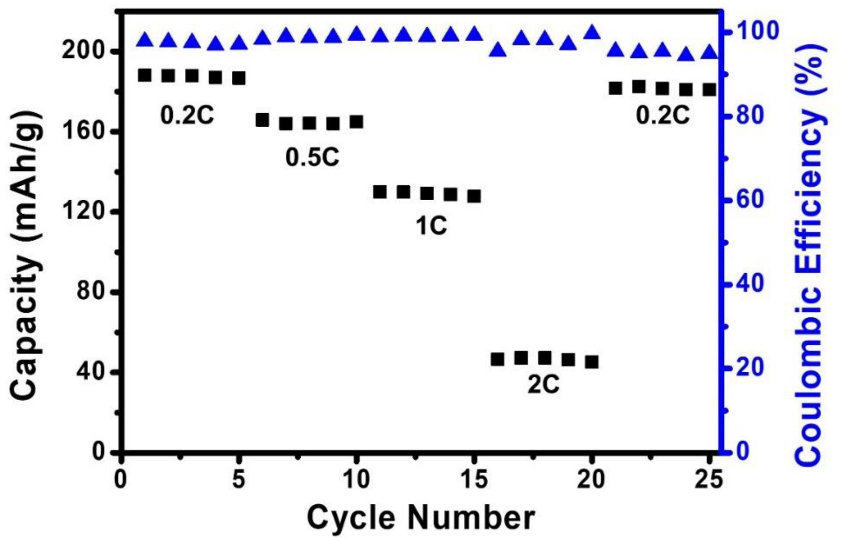

Figure S23. Rate performance of the Li|eutectic PSZ-COF|NCM_{622} full cell at various C-rates.

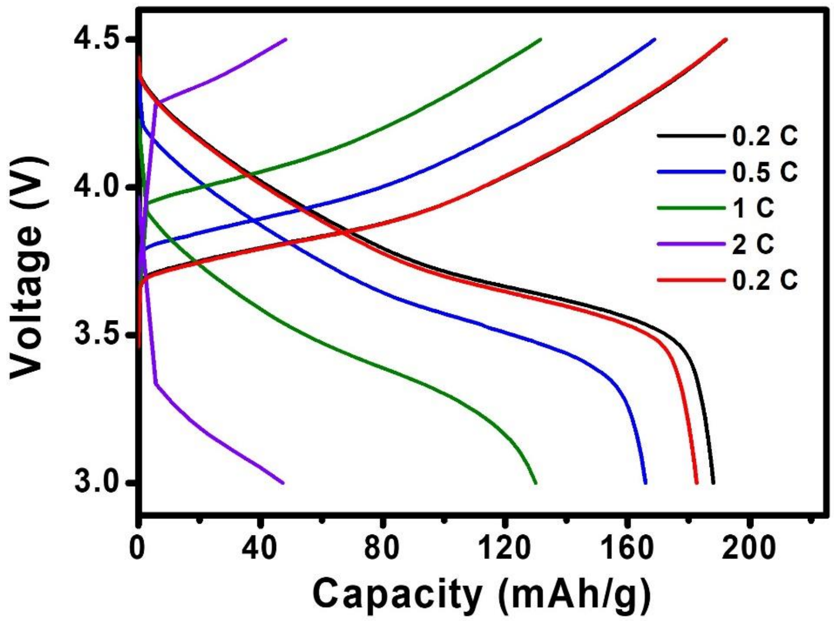

Figure S24. Charge/discharge profile of the Li|eutectic PSZ-COF|NCM_{622} full cell at various C-rates.

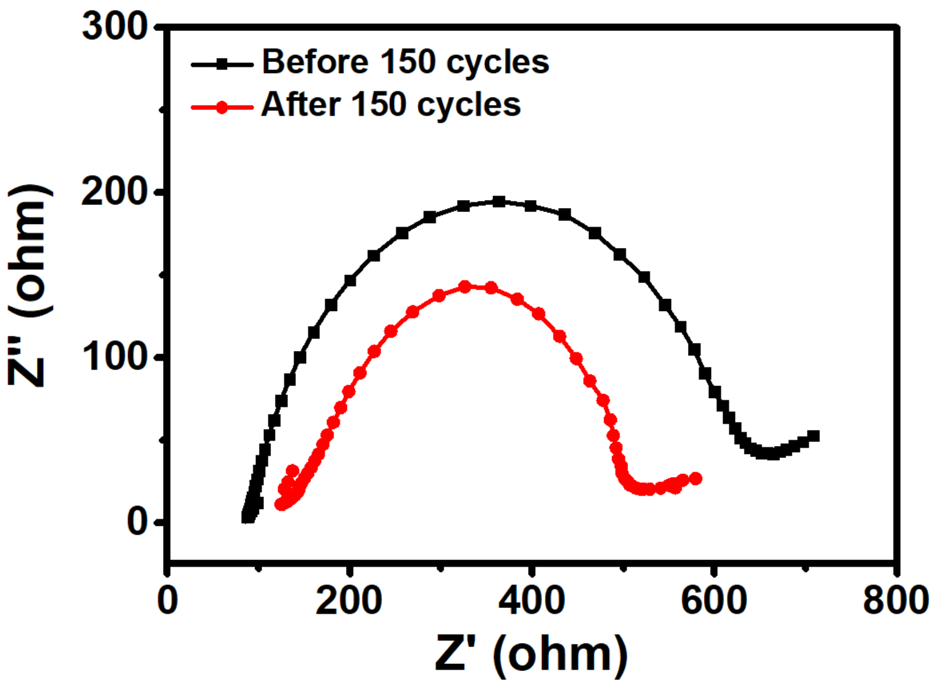

Figure S25. EIS curves for the Li|eutectic PSZ-COF|LFP full cell before and after 150 cycles at 10 mV.

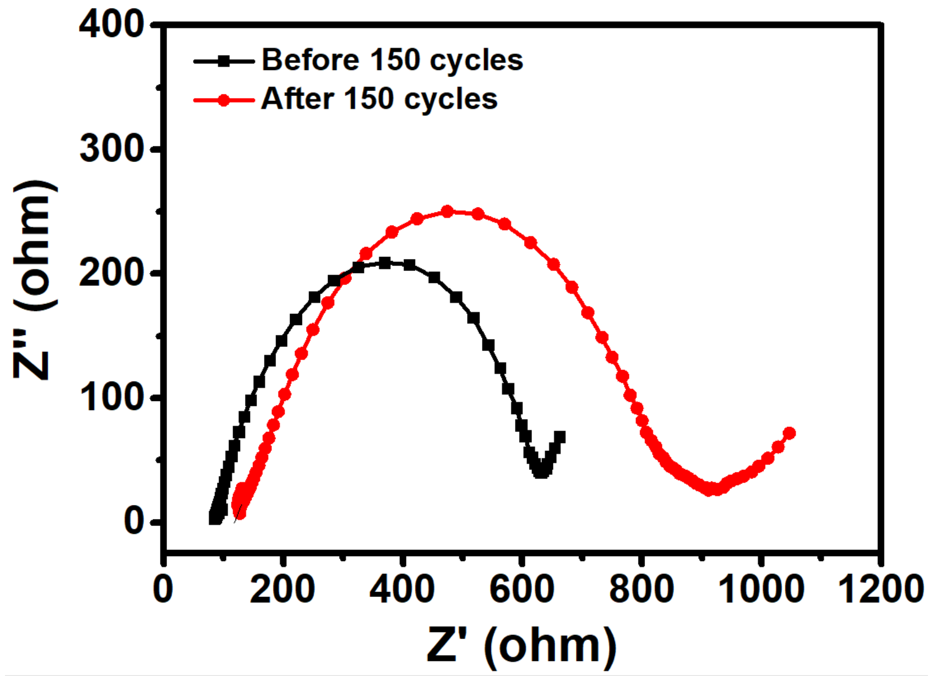

Figure S26. EIS curves of the Li|eutectic PSZ-COF|NCM_{622} full cell before and after 150 cycles at 10 mV.

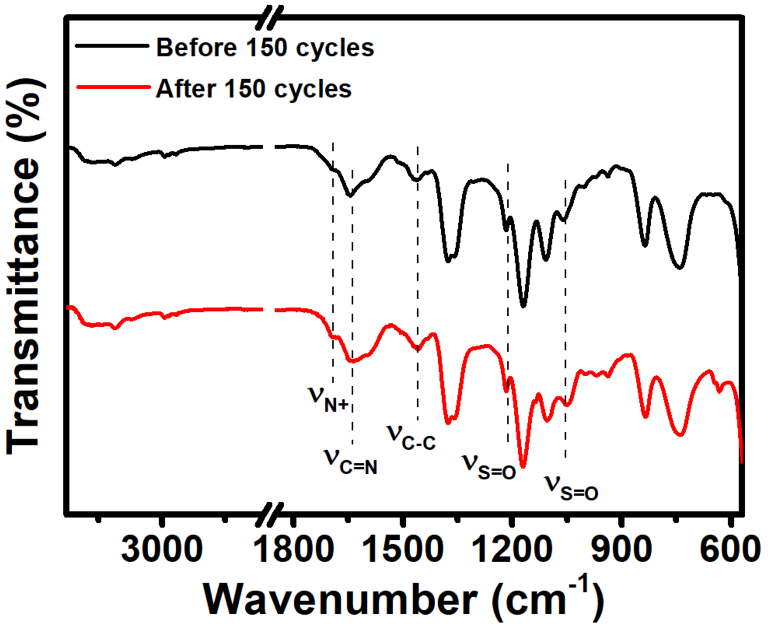

Figure S27. FT-IR spectra of the eutectic PSZ-COF solid electrolyte before and after 150 cycles of the Li|eutectic PSZ-COF|LFP full cell.

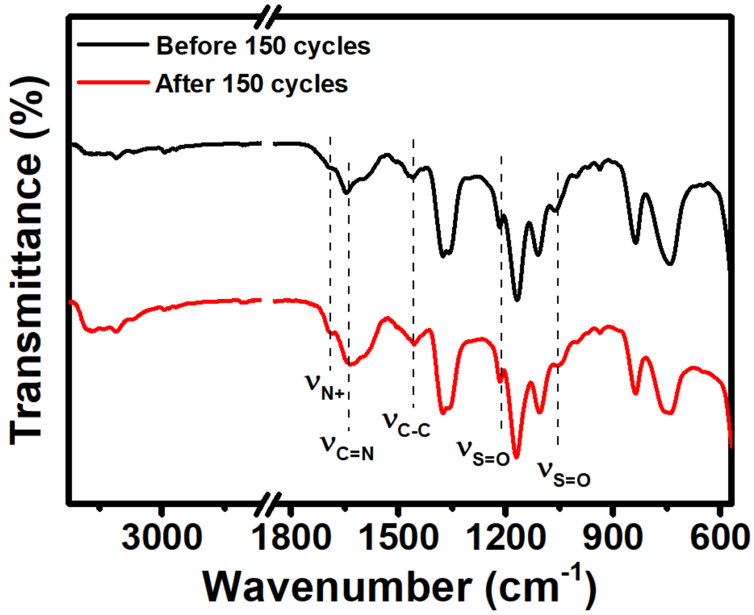

Figure S28. FT-IR spectra of the eutectic PSZ-COF solid electrolyte before and after 150 cycles of the Li|eutectic PSZ-COF|NCM_{622} full cell.

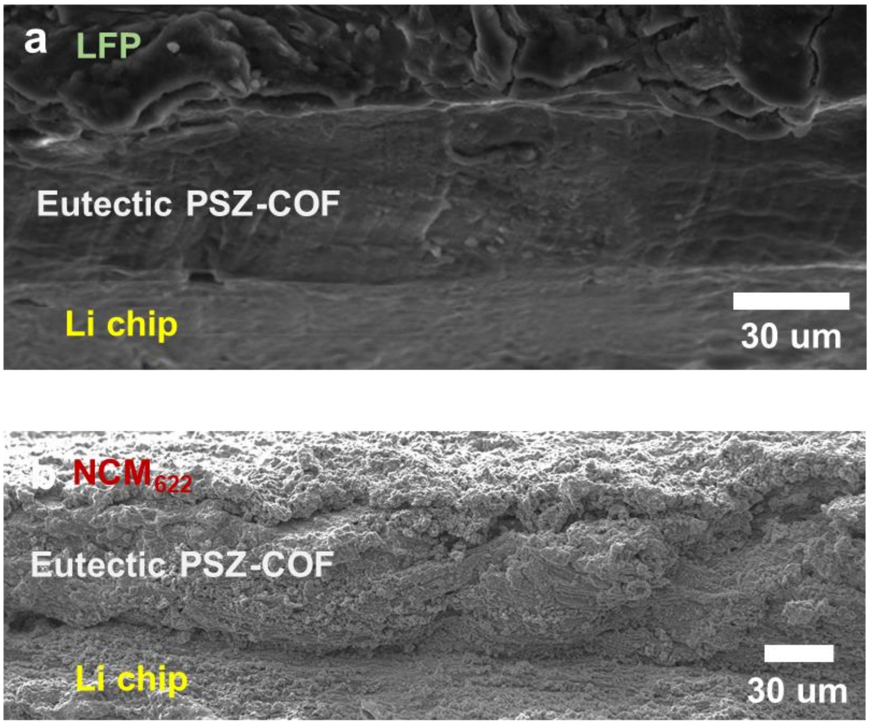

Figure S29. Cross-sectional SEM images of the eutectic PSZ-COF solid electrolyte after 150 cycles of **a)** Li|eutectic PSZ-COF|LFP and **b)** Li|eutectic PSZ-COF|NCM_{622} full cells.
